# Lysozyme-Responsive Hydrogels of Chitosan-Streptomycin Conjugates for the On-Demand Release of Biofilm-Dispersing Enzymes for the Efficient Eradication of Oral Biofilms

**DOI:** 10.1021/acs.chemmater.4c02014

**Published:** 2024-09-30

**Authors:** María Luisa Del Pozo, Antonio Aguanell, Eduardo García-Junceda, Julia Revuelta

**Affiliations:** BioGlycoChem Group, Departamento de Química Bio-Orgánica, Instituto de Química Orgánica General, CSIC (IQOG-CSIC), Juan de la Cierva 3, Madrid 28006, Spain

## Abstract

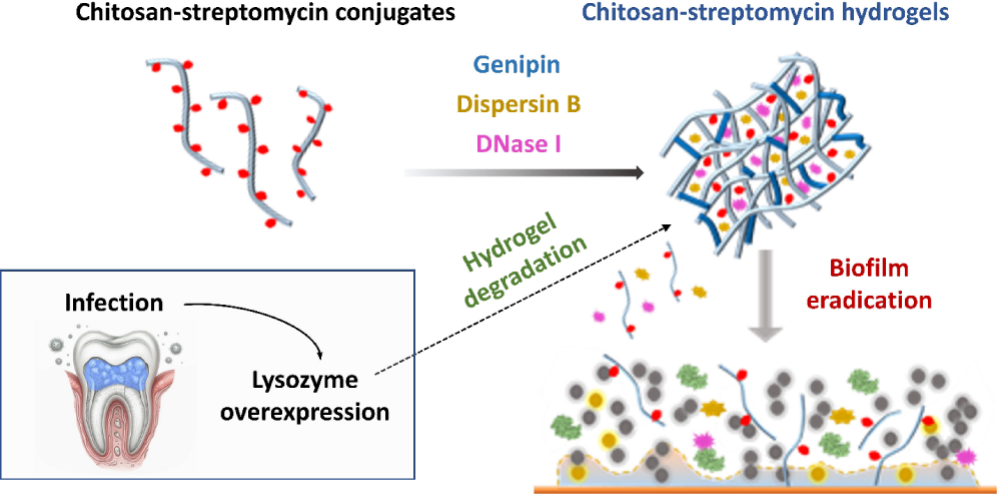

Hydrogels with controlled degradation and sustained antibiofilm activity are promising biomaterials for the treatment of oral infections such as periodontitis or caries. In this article, an *in situ* forming chitosan-streptomycin hydrogel is developed that can target established bacterial biofilms in response to lysozyme, an enzyme that is overexpressed in saliva during oral infections. When the new hydrogel is applied to simulated oral biofilms, the overexpressed lysozyme degrades the hydrogel and releases chitosan-streptomycin oligosaccharides that can eradicate the biofilm. This work has shown that the coupling of chitosan and streptomycin can have a synergistic effect and that the new hydrogel based on chitosan-streptomycin conjugate can effectively combat biofilms of *E. coli*, *S. aureus*, and *P. aeruginosa* formed *in vitro* achieving a significant reduction in the biomass of the biofilm and a substantial reduction in the population of viable bacteria in established biofilms. Finally, the CS-Str hydrogel loaded with biofilm-disrupting enzymes, in particular, DNase I and/or DspB, showed a significantly increased ability to reduce the biofilm biomass of *P. aeruginosa* and *S. aureus* (by over 84% and up to 92%, respectively), resulting in a drastic reduction in cell viability, which fell below 4% for *P. aeruginosa* and below 5% for *S. aureus*.

## 1 Introduction

Oral diseases such as caries and periodontitis are directly linked to the ability of bacteria to form biofilms.^1^ These biofilms are resistant to antimicrobial agents, as they hinder the penetration of antibiotics. In addition, the increasing microbial virulence and antibiotic resistance in oral biofilms or dental plaque have made the prevention and treatment of pathogenic oral biofilms a significant global challenge.^2^ Due to the complexity of the oral cavity and the rapid excretion of substances via saliva, topically applied antimicrobial agents do not maintain the required concentration long enough and do not adhere to the tooth surface to prevent further biofilm formation. When antimicrobials are mixed with planktonic cells, all cells are exposed to the full dose,^3^ while the biofilm matrix may restrict access and prevent penetration of the agents into the extracellular polymeric substances (EPSs). Finally, the acidic pH characteristic of oral biofilms reduces the efficacy of many antibiotics.

Although progress has been made in understanding the mechanisms of biofilm formation and their persistence, there are still few new and effective treatment options. In this sense, it is very worrying that, on average, less than one new antibiotic is brought to the global market each year.^4^ This is due to the significant costs associated with the development and approval of new drugs, as well as the low financial returns generated from the sale of antibiotics.^5^ In this context, the available agents for the treatment of oral biofilms are limited to broad-spectrum antimicrobials such as chlorhexidine,^3^ which is not suitable for long-term daily use due to its negative effects (tartar formation and tooth discoloration).^6^

Enzymatic dispersion of biofilms is a new field of research, and enzymes such as glycoside hydrolases, proteases, or deoxyribonucleases have been shown to effectively disperse biofilms and convert colonized microbial cells to a more susceptible planktonic state, resulting in increased susceptibility to antibiotics and the host’s immune system.^7,8^ However, the therapeutic use of these enzymes in the clinic is associated with significant limitations, such as their high susceptibility to degradation and rapid loss of activity when exposed to unfavorable environmental conditions.^9^

Existing antibiotic formulations and derivatization offer an alternative approach to solving this problem by reducing the time and cost of developing new therapies while offering several improvements over current therapies by increasing the drug efficacy and reducing unwanted side effects. This is particularly true for bacterial biofilms, where drug delivery can improve the diffusion of antibiotics through the exopolymeric matrix and prevent their premature deactivation by preventing the binding of drugs to matrix components and their enzymatic deactivation. Among the numerous approaches developed, polymeric systems have unique advantages such as their flexible structural design, processability and recyclability, tunable platform construction, and safety, enabling their fine-tuning to achieve optimal drug activity.^10^ Polymeric systems can deliver their selected drug cargo either by encapsulation^11^ or conjugation in a polymer matrix^12^ or by therapeutic conjugation to a fully soluble polymer chain with a single backbone.^13^ A major advantage of the latter is their flexible, random coil structure, which allows them to easily penetrate gaps smaller than their hydrodynamic diameter.

Chitosan (CS), a semisynthetic polysaccharide derived from chitinous biomass, has remarkable potential in this regard, not only because of its biocompatibility, biodegradability, and low toxicity, but also because of its inherent antibacterial activity.^14^ Chitosan has a positive charge that attaches to the cell walls of bacteria and helps to kill or inhibit them. Chitosan is also effective against biofilms, and the ability of chitosan to destroy alredy formed biofilms has been documented.^15^ Combined with its ability to form a film that adheres to the tooth and protects it from plaque, this makes chitosan a suitable polymer for dental applications.^16^ In addition, its chemical modification can produce new and attractive physicochemical properties compared to the original chitosan, as well as interesting pharmacological properties and biological activities.^17,18^ In our previous studies, we have developed hybrid hydrogels of sulfated chitosan and lysozyme that can be customized to degrade at different rates and have a sustained antibacterial effect.^19^ These antibacterial films not only maintained the activity of lysozyme but also enhanced its antimicrobial effect due to a possible synergistic effect between lysozyme and chitooligomers formed after the degradation of chitosan hydrogels.

Another interesting strategy for modifying chitosan is to bind bioactive substances to the polymer chain via covalent bonds. In particular, chitosan-antibiotic conjugates have proven to be an effective form for improving the therapeutic and biological effects of conventional antibiotics. For example, several studies have shown that chitosan can be used as a covalent carrier to deliver antibiotics, such as streptomycin, into biofilms.^20−22^ However, the rapid excretion of these antibacterial agents via saliva may limit their use in antibiofilm therapy. In this regard, the sustained release of these polymer-drug conjugates from a reservoir could be an effective and useful method to overcome this drawback.

**Scheme 1:**
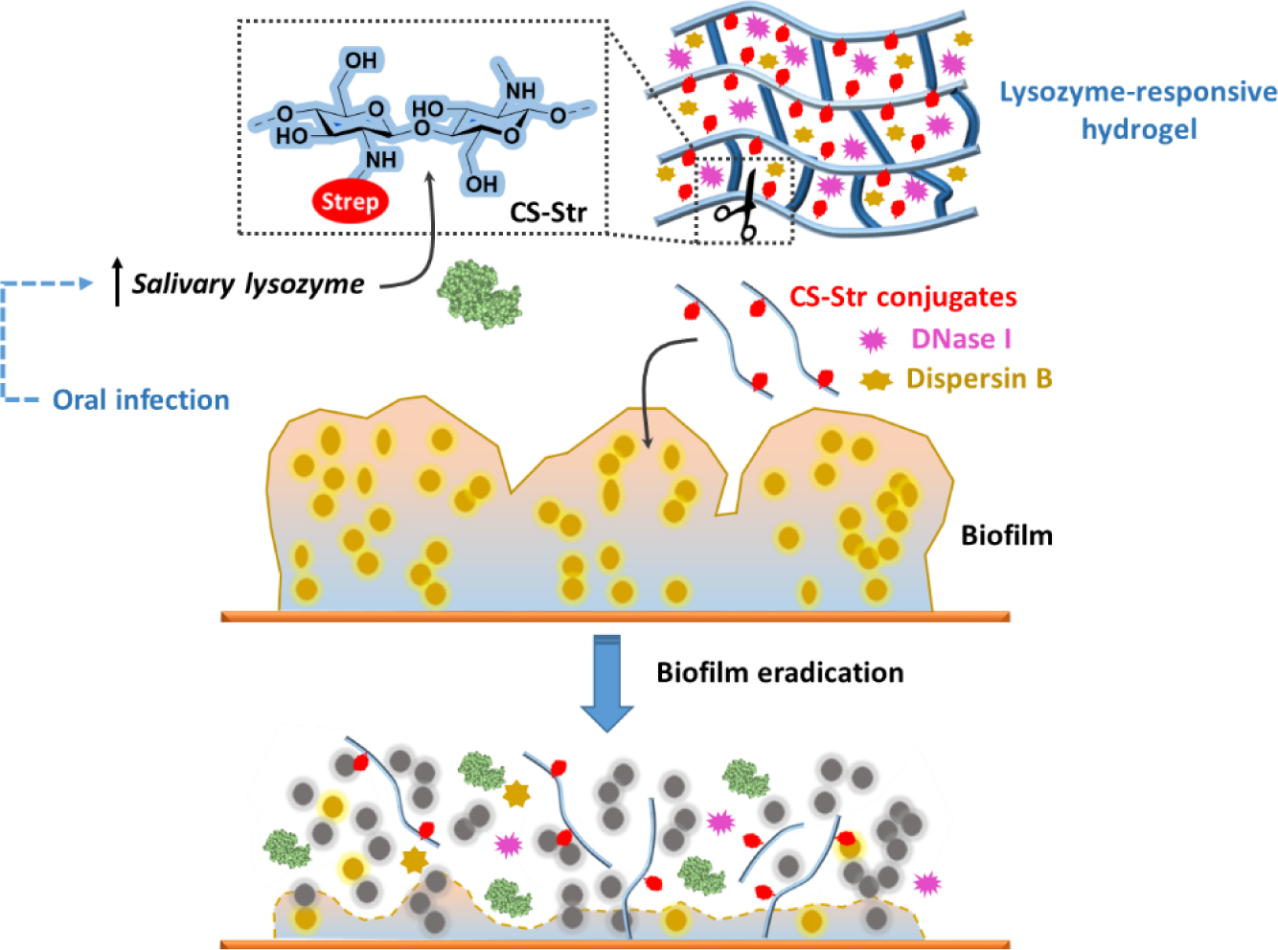
Schematic Representation of the CS-Str Hydrogel and its Anti-Biofilm Mechanism Due to the overexpression of lysozyme in response to oral biofilm infection, the glycosidic bonds of CS-Str are hydrolyzed. This enables the on-demand release of CS-Str oligosaccharides and biofilm-disrupting enzymes that can eliminate bacterial biofilms.

To achieve this goal, a novel hydrogel based on chitosan-streptomycin conjugates with successive responses to lysozyme is developed. Lysozyme is a salivary protein that, under normal conditions (at an approximate concentration of 2 μg/mL), contributes to the establishment and maintenance of a stable ecosystem in the oral cavity, in which harmless species predominate over potentially dangerous species and provide microbial protection. In response to oral biofilm infection, this antibacterial enzyme is overexpressed in saliva at concentrations of >10 μg/mL to control microbial overgrowth, reduce the number of bacteria in the dental biofilm, decrease colonization, and alter bacterial metabolism. Accordingly, when the responsive hydrogels developed in this work are applied to biofilm-infected areas, lysozyme would induce matrix cleavage, degradation of the hydrogel, and release of CS-Str fragments to eradicate biofilm areas (Scheme 1).

Once the bacteria are killed, the lysozyme concentration may be recovered to normal physiological conditions, and the drug release slows, resulting in an on-demand drug release manner. In this way, the hydrogels serve as a platform of bioactive oligosaccharides that provide spatial and temporal control over the release of therapeutic chitosan. Benefiting from this controlled degradation hydrogel demonstrated great promise in the on-demand corelease of bioactive oligosaccharides and biofilm dispersal enzymes such as DNase I and/or Dispersin B (DspB).

## 2 Experimental Section

### 2.1 Materials

Chitosan (CS) (degree of deacetylation 95%; molecular weight 50 kDa) was purchased from Glentham Life Science Ltd. (United Kingdom). All reagents were commercially available and were used without further purification.

### 2.2 Synthesis of Chitosan-Streptomycin Conjugates

CS-Str conjugates were prepared according to Li et al.^22^ Briefly, 500 mg of CS were suspended in 10 mL of distilled H_2_O, and the mixture was magnetically stirred at 35 °C for 24 h; then 10 mL of 2 M AcOH in distilled H_2_O was added to the mixture. Then, Str (0.7, 1.0, 2.2, or 3.5 g) dissolved in 10 mL of distilled H_2_O were mixed with the chitosan solution. The reaction was left under magnetic stirring for 1 h, after which 5 mL of 1.8 M NaBH_3_CN in distilled H_2_O was added, and the reaction mixture was left under magnetic stirring at 35 °C for 24 h. The mixture was then neutralized with 2 M NaOH in distilled H_2_O, extensively dialyzed against distilled H_2_O in a membrane with 14 kDa pore size, and finally freeze-dried. The overall degree of streptomycin incorporation (DS) was determined by comparing the ^1^H NMR integrals of the protons of the methyl group of the streptomycin molecule (1.22 ppm) with those of the acetyl group of the chitosan molecule (2.01 ppm).

### 2.3 Hydrogel Preparation

Hydrogels were prepared according to the conditions described by Heimbuck et al.^23^ Briefly, 200 mg of CS or 300 mg of CS-Str were dissolved in 10 mL of 0.075 M AcOH in distilled H_2_O and magnetically stirred for 24 h. CS or CS-Str was cross-linked with genipin at different concentrations: 1, 1.5, 2, or 3% weight (wt), with respect to chitosan. For this purpose, a genipin stock solution was prepared by dissolving genipin powder in 100% ethanol at a ratio of 5 mg/mL and then adding the desired final volume of genipin to the chitosan solution, which was magnetically stirred for 30 min to form the hydrogel precursor solution. After this time, the precursor solution was sonicated for 30 min to promote homogeneous mixing and remove air bubbles from the medium. The solution was then poured into a 6 cm diameter glass Petri dish previously coated with a Sigmacote solution and incubated at 50 °C for 24 h in an oven for homogeneous heating. Absorbance spectral scans in the visible range (300–800 nm) were used to monitor the rate of cross-linking throughout the reaction by monitoring the intensity of the 610 nm peak.

### 2.4 Gelification of Chitosan by Genipin and β-Glycerophosphate

For this purpose, the conditions of Maiz-Fernández et al.^24^ were followed, with minor modifications. 1.18 mmol of the substrate to be cross-linked was dissolved in 10 mL of 0.075 M AcOH in distilled water and was kept under magnetic stirring for 24 h. Subsequently, 566, 849, or 1132 mg of β-glycerophosphate previously dissolved in 2 mL of distilled water were added dropwise under magnetic stirring. Once the gelation additive has been added, the genipin solution is added and proceeded as previously described in the formation of hydrogels.

### 2.5 Ninhydrin Assay

The ninhydrin assay was used to measure the concentration of unreacted amines on the chitosan in chitosan hydrogels cross-linked with genipin as a function of the genipin concentration in the precursor solution.^23^ A d-glucosamine stock solution of 50 μM in 0.05% (v/v) glacial acetic acid was used to prepare the standard curve. Hydrogels (2 mg) cross-linked with genipin (1, 1.5, 2, or 3 wt %, with respect to CS or CS-Str) were swollen overnight in 0.5 mL of water. Ninhydrin reagent (0.5 mL) was then added to the hydrogel samples and heated to 100 °C in a water bath for 10 min. The solution was then cooled to room temperature, centrifuged at 16000 *g* for 5 min, and diluted 10 times using 95% ethanol. The absorbance at 570 nm, proportional to the number of free amine groups present in the sample, was recorded. The concentration of free amines in each group was calculated using the d-glucosamine standard curve. The cross-linking degree (CD) was calculated using eq 1:
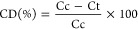
1where Cc is the moles of free amines in the control chitosan and Ct is the amine group concentration in the cross-linked hydrogels.

### 2.6 Monitor the Rate of Chitosan–Genipin Cross-Linking

The gelation kinetics of the different synthesized hydrogels was evaluated by monitoring the absorbance in the visible range (300–800 nm) also following the conditions of ref (23), by monitoring the intensity of the 610 nm peak. The absorbance spectra of the sample were measured at the beginning of the cross-linking reaction (0 h) and during the reaction at 50 °C at different time intervals (10, 20, 30, 40, 50, 50, 60, 90, 90, 120, 180 min and 24 h). For this purpose, cuvette gelation was performed, and the same versions of the precursor solution of the hydrogels without genipin were used as blanks.

### 2.7 Gel Fraction

The gel fraction was evaluated for chitosan and chitosan-streptomycin hydrogels at 1.5 and 2% genipin, respectively. For this purpose, 1.18 mmol of the substrate of interest was taken, the protocol for the preparation of hydrogels was followed, and the gel fractions obtained were 84% for chitosan and 89.6% for chitosan-streptomycin.

### 2.8 Swelling

The swelling ratio of the xerogel was determined by a gravimetric method.^25^ The stored hydrogel disks were weighed (Wd) and then immersed in 10 mL of salivary medium with different pH values: physiological and at the average pH of the biofilms (pH = 5.5) for 24 h at 25 °C, and then weighed again (Ws). Finally, the swelling ratio was quantified using eq 2:
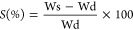
2

### 2.9 Salivary Medium Formulation

For the preparation of the in vitro salivary medium, the formulation indicated by Tonguc Altin et al.^26^ was followed. To do this, first the inorganic medium of the formulation must be prepared, 128.6 mg of NaCl, 3.2 mg of MgCl_2_·6H_2_O, 59.8 mg of CaCl_2_, and 29.8 mg of KCl are dissolved in 100 mL of distilled H_2_O. Subsequently, 1 mL of the inorganic medium is taken, and 472 μL of 85% (w/v) H_3_PO_4_ and 2 mg of methylcellulose are added. Once the methylcellulose is dissolved, the solution is neutralized with a 1% (w/v) KOH solution in distilled water to pH 5.5 or 7.0 depending on the final use.

### 2.10 Evaluation of the Degradation of Hydrogels in Salivary Medium In Vitro

Degradation of CS and CS-Str hydrogels in salivary medium was analyzed using a gravimetric method by evaluating the difference in weight after incubation with the lysozyme. To do this, the hydrogels were previously dried at 50 °C for 48 h; once dried, the assay was initiated by adding 1 mL of salivary medium containing 10 μg/mL of lysozyme to the xerogels. Every 24 h for 4 days, the supernatant was removed for further treatment, and the lysozyme solution was renewed. The supernatants were heated for 10 min at 100 °C before treatment in order to denature the lysozyme. The change in dry weight was quantified using eq 3:
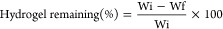
3where Wi and Wf indicate the dry weight at the beginning and at the end of the experiment.

### 2.11 Quantification of Reducing Ends

The determination of reducing ends in the salivary medium after the degradation of hydrogels study was performed by a colorimetric assay using 3,5-dinitrosalicylic acid (DNS) as the oxidizing agent. A sample of 250 μL of each supernatant were lyophilized and then dissolved in 50 μL of distilled water. After this, 50 μL of DNS solution was added and heated for 8 min at 100 °C and its Abs value was measured at λ = 540 nm.

### 2.12 Rheological Analysis

The gel point was determined by rheology at 37 °C. First, the determination of the linear viscoelastic region LVER was carried out to ensure that the subsequent test was within the region where stress and strain are proportional and insufficient to cause deformation in the hydrogel structure, working then with a stress whose order of magnitude was below the critical stress, i.e., a stress of 10%. For this purpose, an ARG2 rheometer (TA Instruments, UK) with a 20 mm diameter parallel plate geometry and Peltier with temperature control at 37 °C was used. First, a stress or strain sweep test was carried out on an already formed hydrogel to determine the linear viscoelastic region (LVER). For this purpose, the hydrogel was positioned on the Peltier, and the upper geometry was aligned in such a way that it would be in close contact with the hydrogel without breaking it (50 μm separation with respect to the Peltier). The scanning was done in a range of oscillation voltages from 0.01 to 1000%, at a constant temperature of 50 °C and a frequency of 1 Hz. Next, for the gelation versus time test, 500 μL of the precursor solutions of the chitosan-streptomycin and chitosan-streptomycin hydrogels plus566 mg of G2P were added on top of the Peltier preheated to 37 °C. The upper geometry was adjusted to a distance of 50 μm from the Peltier, and a 10% voltage was applied at a frequency of 1 Hz following the gelation kinetics for 3 h (10 800 s).

### 2.13 Biofilm Formation

*E. coli* K12. *P. aeruginosa* (NCIMB 8295) and *S. aureus* (NCTC 8532) biofilms were cultured in 24-well plates according to the method of Danese et al.^27^ with slight modifications. Briefly, a previously isolated colony was inoculated into 5 mL of LB medium and incubated for 24 h at 37 °C and 120 rpm. 200 μL of the overnight grown culture containing (1–5) × 10^5^ CFU/mL was added to a 24-well plate that was incubated at 37 °C for 24 h. The next day, the wells were checked for biofilm formation. The culture medium was removed, and the wells were washed with 300 μL of 0.9% saline medium. The plate was then dried at 35.5 °C for 20 min. The wells were then stained with 300 μL of 1% crystal violet for 15 min. The dye was then removed, and 4 washes were performed with 300 μL of water each. Finally, the plates were dried at room temperature.

### 2.14 Biofilm Disruption Assay

The effect of chitosan (CS) and streptomycin-conjugated chitosan polymers (CS-Str) and the corresponding hydrogels on the preformed biofilm was determined. Briefly, the hydrogels were loaded with lysozyme (10 mg/mL) using a method previously optimized in our laboratory. The polymers and hydrogels were either loaded with lysozyme (10 mg/mL), for which a method previously optimized in our laboratory was used,^19^ Alternatively, the polymers and the hydrogels were treated with artificial saliva containing or not containing lysozyme at a concentration of 10 μg/mL. The 24-well plates containing biofilms were washed three times with 300 μL of 0.9% sterile saline medium to remove planktonic bacteria and then air-dried for 20 min. Then, in the case of CS and CS-Str polymers, 500 μL of a solution of the different polymers was added to the wells at a concentration of 250 μg/mL. In the case of the hydrogels, xerogel disks (ø= 2 cm) were placed directly on the biofilm and incubated for 24 or 48 h as indicated. After the incubation period, either the supernatants or the hydrogels were carefully removed, and the wells were stained with 1% crystal violet solution as previously described. The biofilm was then dissolved with 1 mL of absolute ethanol for 24 h shaking at 140 rpm so that the biofilm mass was dispersed in the solution, which was carefully transferred to a cuvette to perform absorbance measurements at 570 nm.

### 2.15 Bacterial Viability in Preformed Biofilms

Living bacteria within mature biofilms treated with CS or CS-Str hydrogels were quantified by the colony count method.^28^ Cell survival was studied with CS and CS-Str hydrogels loaded with a 10 mg/mL lysozyme solution and with the unloaded gels in artificial saliva containing 10 μg/mL lysozyme. The gels were placed directly on the biofilms in a multiwell plate and, if indicated, 200 μL of the lysozyme solution or artificial saliva was added. As a control, 300 μL of a 250 μg/mL solution of lysozyme was added to the biofilm. After 24 h of incubation, the hydrogels and control solutions were removed, and after washing the biofilms twice with water, 1 mL of LB medium was added and the plates were shaken at 140 rpm and 37 °C for 24 h. After this time, a series of serial dilutions of the overnight grown cultures were performed. 100 μL of the final dilution was seeded onto LB agar plates. The plates were incubated at 37 °C for 24 h, after which the number of colony forming units (CFU) per plate was counted.

### 2.16 Cytotoxicity Evaluation Against A549 Cells

A549 cells were cultured with Dulbecco’s modified Eagle’s medium (DMEM) supplemented with 10% v/v fetal bovine serum and 1% v/v penicillin-streptomycin (10,000 U/mL) at 37 °C and 5% CO_2_. Cells were then seeded in a standard 96-well flat-bottomed cell culture plate at a density of 5 × 10^3^ cells/mL and incubated with 5% CO_2_ at 37 °C for 24 h to allow adhesion. Culture medium was then removed from each well and replaced with 200 μL of either culture medium alone (positive control) or serial dilutions of hydrogel extracts obtained by incubating 5 mm of a diameter fragment of set hydrogel in 2.5 mL of saliva medium containing Lyz 10 μg/mL, for 48 h at 37 °C and stirring at 50 rpm. When indicated, a 5 mm diameter fragment of the hydrogel was employed in place of the hydrogel extract. The masses of the hydrogels used were 0.4 mg for both CS and CS-Str, and 1.3 and 1.5 mg for CS/G2P 1.87 M and CS-Str/G2P 1.87 M, respectively.

The 96-well plate was then placed in an incubator for an additional 48 h. Following incubation, 20 μL of a 0.05% resazurin working solution per well was added, and the plates were further incubated for 3 h. Optical density (O.D) at a wavelength of 590 nm was measured, and the percentage cell viability (%) was calculated using eq 4:^29 −34^
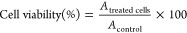
4

where *A*_trated cells_ represents the mean of absorbance in the cells treated with the tested hydrogel and *A*_control_ denotes the mean of absorbance of the untreated cells. All assays were performed in triplicate, and the results were obtained from three independent experiments.

## 3 Results and Discussion

### 3.1 Chitosan and Chitosan-Streptomycin Conjugates as Building Blocks for Antibiofilm Hydrogel Development

#### 3.1.1 Synthesis and Characterization of Chitosan-Streptomycin Conjugates

Since the enzymatic degradation of the hydrogel is crucial for achieving the desired therapeutic results, an analysis of the degradation rate of different CSs with Lyz was performed prior to the synthesis of chitosan-streptomycin conjugates (see Supporting Information).

**Figure 1:**
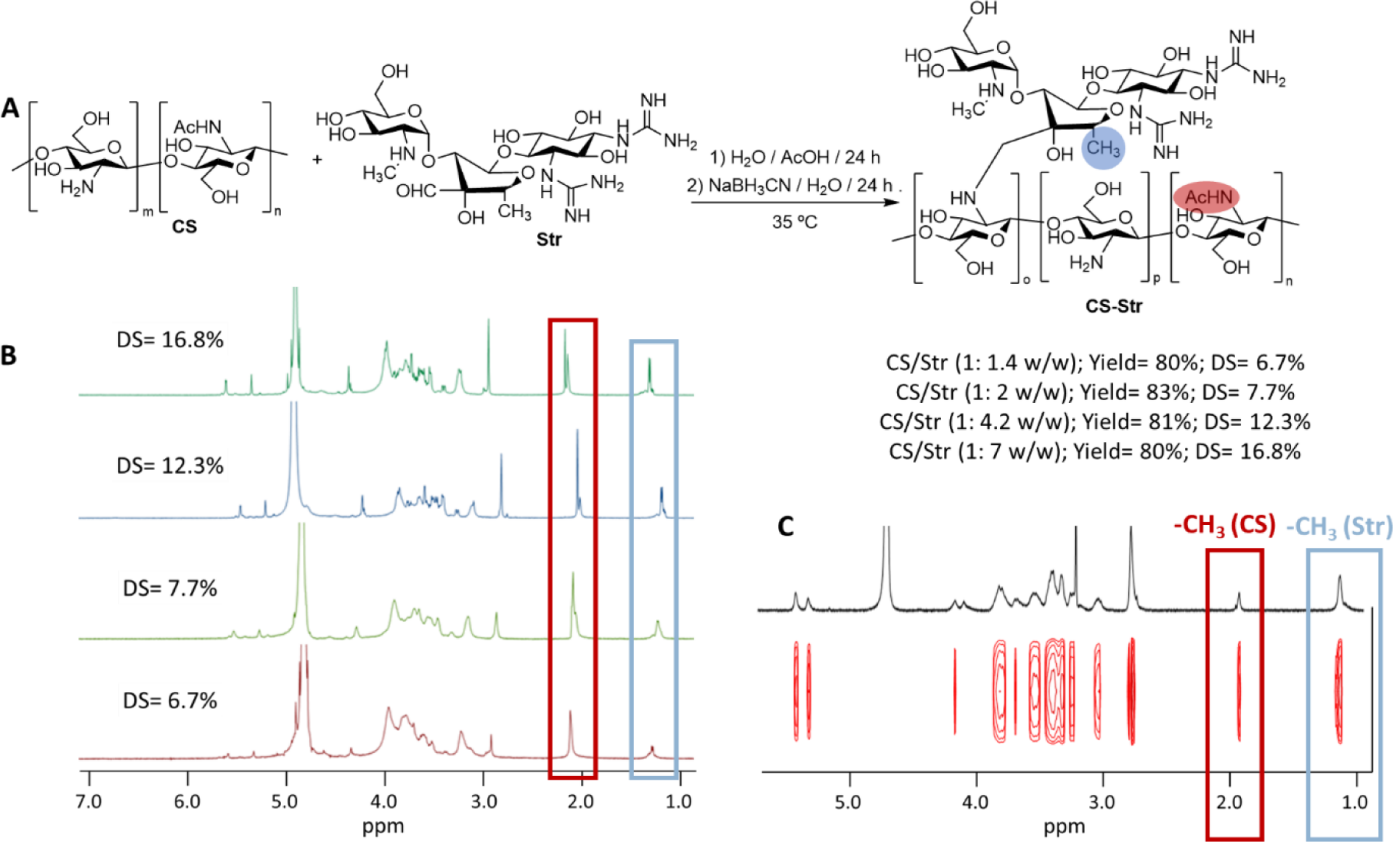
Preparation and Characterization of CS-Str conjugates. A) Synthesis scheme for the preparation of chitosan-streptomycin conjugates (CS-Str). B) ^1^H NMR spectra of four CS-Str conjugates obtained, showing methyl group peaks of streptomycin at about 1.22 ppm (blue boxes) and methyl group peaks of chitosan at about 2.01 ppm (red boxes). Integration of these peaks allowed us to determine the overall degree of streptomycin incorporation (DS). C) DOSY spectrum of CS-Str with a degree of streptomycin incorporation of 16.8%. Conjugation of CS and Str was evaluated by DOSY-NMR experiments to exclude the possibility of a simple physical mixture of CS and Str. Both CS and streptomycin have the same translational diffusion coefficient in D_2_O, suggesting that they are linked by a covalent bond.

The chitosan-streptomycin conjugates (CS-Str) were prepared according to a previously described procedure based on the formation of a Schiff base between the amino groups of chitosan and the aldehyde groups of streptomycin, followed by the reduction of the resulting imine (Figure 1A).^20^

The efficiency of the conversion was evaluated by ^1^H NMR. The disappearance of the signals at 9.77 ppm (streptomycin-aldehyde) and 5.06 (streptomycin-hemiacetal-aldehyde) and the presence of streptomycin signals such as the methyl groups at 1.22 proves the reaction coupling (Figure S2). The degree of substitution achieved as a function of the w/w ratio of CS/Str was estimated by comparing the ^1^H NMR integrals of the protons of the methyl group of the streptomycin molecule (1.22 ppm) to the protons of the acetyl group of the chitosan molecule (2.01 ppm) (Figure 1B). Finally, the CS-Str conjugates were analyzed using diffusion order spectroscopy (DOSY-NMR), a spectroscopic method that distinguishes compounds based on their respective translational diffusion coefficients. These experiments showed that the streptomycin was covalently bound to the CS chain (Figure 1C).^25^ Indeed, both streptomycin and the CS chain exhibited the same translational diffusion coefficient at 25 °C in D_2_O, although they differed significantly in molecular weight. CS-Str with a degree of substitution of 16.8% (hereafter CS-Str) was chosen for the rest of the work, as the antibiofilm properties of chitosan-streptomycin conjugates depend on the streptomycin concentration.

#### 3.1.2 Analysis of Antibiofilm Effect of CS and CS-Str

**Figure 2:**
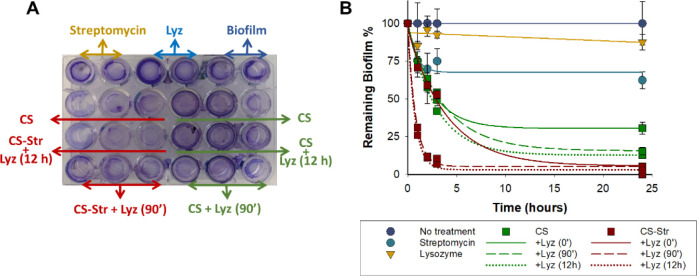
Quantification of the bacterial biofilm. A) Photograph showing formed *E. coli* biofilm and remaining biofilm after 3 h of incubation with polysaccharides in the presence of Lyz and with the mixtures obtained after incubation of polysaccharides with Lyz for 90 min and 12 h. B) Percentage of remaining biofilm after incubation with Lyz, Str, and polysaccharides (without and with previous incubation with Lyz); values are the average of three replicates, and standard deviations are shown.

The ability of chitosan and its streptomycin conjugates to damage biofilms formed by bacteria has been documented, but is largely dependent on the structural properties of the polysaccharide, such as molecular weight,^35^ degree of *N*-deacetylation,^36^ or degree of streptomycin conjugation.^20^ For this reason, before preparing the hydrogel to see if CS and CS-Str conjugate are able to destroy the bacterial biofilms formed by *E. coli*, the antibiofilm effect of CS and CS-Str was evaluated. To simulate the mode of action of the hydrogel, we investigated not only the effect of the starting material CS and the conjugate CS-Str in the presence of Lyz but also the mixture obtained after incubation of these polysaccharides with Lyz for 90 min and 12 h before addition to preformed biofilms. The loss of biofilm biomass was determined by the crystal violet staining assay (Figure 2).^37^

After 24 h, Lyz and Str alone had no significant effect and 90% and 60% of the biofilm remained, respectively. A combination of CS with Lyz improved biofilm reduction, an effect that was enhanced after prior incubation of the polysaccharide with the enzyme. Thus, while 30% of the biofilm remained without incubation, this value decreased to 15 and 12% with prior incubation (90 min and 12 h, respectively). It is noteworthy that when the conjugate CS-Str was used, complete (or quasi-complete) destruction of the biofilm was observed after 24 h of exposure. In this case, prior incubation of the conjugate with Lyz accelerated the removal of the biofilm, leaving only 10% of the biofilm after 3 h. These results confirm not only that the conjugation of streptomycin to chitosan efficiently damages established biofilms^20^ but also that this activity is strongly influenced by the degree of polymerization of the chitosan.^22^

### 3.2 Chitosan- and Chitosan-Streptomycin Hydrogels as Platforms with Fine-Tune Degradability and Sustained Inherent Antibiofilm Activity

#### 3.2.1 Preparation and Characterization of Hydrogels

**Figure 3:**
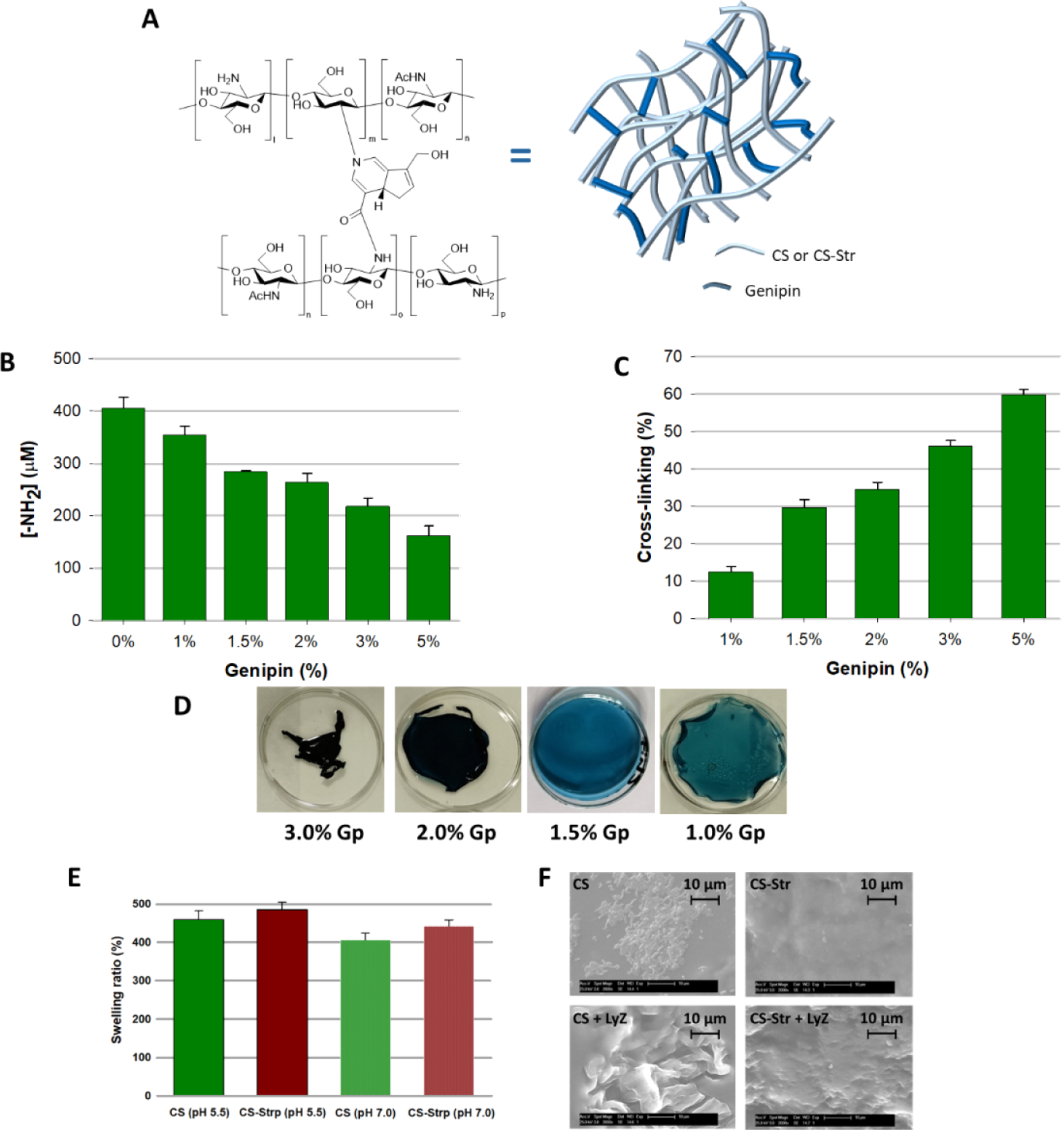
Preparation and characterization of the hydrogels. A) Molecular structure of chitosan molecules cross-linked with Gp (left) and schematic representation of chitosan hydrogel networks formed by chemical cross-linking (right). B) Reactive amine concentration quantified using the ninhydrin assay and C) cross-linking degree for different hydrogel formulations. D) Overall view of chitosan hydrogels cross-linked with different Gp concentrations. E) Swelling ratio of hydrogels calculated by the ratio of wet and dry weights of hydrogels for 48 h at different pH values (5.5 and 7.4). F) Morphological observations of CS (left) and CS-Str hydrogels (right) by cryo-SEM at days 0 (left) and 4 (right) in saliva medium with Lyz. In B,C, and E, values are the average of three replicates, and standard deviations are shown.

Among the various methods for preparing chitosan hydrogels by chemical cross-linking described previously,^38^ chitosan hydrogels cross-linked with genipin (Gp) were prepared in the present study.^39^ Gp is a natural cross-linking substance found in the fruits of *Gardenia jasminoides*. It is 10 000 times less cytotoxic than glutaraldehyde, but can be used to produce cross-linked materials with comparable properties.^40,41^ Under acidic conditions, Gp behaves like a dialdehyde, similar to glutaraldehyde (Figure 3A), but its condensation products are much more stable than those of glutaraldehyde.

To optimize the preparation process, CS was cross-linked with Gp at different concentrations (1.0, 1.5, 2.0, 3.0, or 5.0 wt %), and the best experimental conditions were determined based on the degree of cross-linking and the integrity of the hydrogel. The ninhydrin assay was used to monitor the cross-linking rate of CS-Gp. This allowed quantification of the concentration of unreacted amines on the chitosan in the genipin cross-linked chitosan hydrogels (Figure 3B).

The difference in free amines in the genipin hydrogels shows an increase in total cross-linking from 12.5% using 1.0 wt % genipin to 59.8% using 5 wt % Gp (Figure 3C). Considering that the hydrogels were designed as platforms with spatiotemporal dynamics due to their lysozyme-mediated degradation, and taking into account that hydrogels with a higher degree of cross-linking degrade more slowly than hydrogels with a lower degree of cross-linking,^19^*in vitro* characterization experiments were performed with chitosan and chitosan-streptomycin hydrogels containing 1.5 and 2.0 wt % genipin cross-linker to minimize the degree of cross-linking and maintain physical integrity (Figures 3D).

For both hydrogels (CS and CS-Str), the highest degrees of swelling were observed at pH 5.5, which could be due to the fact that under these conditions the chitosan amines are protonated, leading to dissociation of secondary interactions such as intramolecular hydrogen bonds, allowing more water to enter the gel network (Figure 3E). A similar effect may be responsible for the higher degree of swelling of the CS-Str hydrogel compared to CS. When chitosan is conjugated with streptomycin, the chitosan amines remain partially deprotonated at pH 7.4, but the guanidine groups of streptomycin are fully protonated, increasing repulsion in the polymer chains and allowing greater swelling.

The *in vitro* stability of xerogels was also investigated by determining the mass loss after incubation in saliva medium with Lyz. The percent degradation values were 21.2 ± 3.5% and 3.5 ± 0.6% for the CS hydrogel and 14.7 ± 2.8% and 1.5 ± 0.3% for the CS-Str hydrogel after incubation in saliva medium with 10 and 2 μg/mL of Lyz (concentration under oral biofilm infection and normal conditions, respectively). In both cases, degradation was attributed to hydrolysis of the glycosidic bonds of the chitosan by Lyz, and the lower degradation of the CS-Str hydrogels may be due to the lower activity of Lyz toward the modified chitosan than the parent chitosan. Indeed, the lytic activity of the lysozyme against the CS hydrogel was higher than that of the CS-Str hydrogel, based on the increase in reducing ends observed in the supernatant after 4 days of incubation of the hydrogel in salivary medium with Lyz (10 μg/mL) (431.5 ± 12.3% increase for CS versus 388.0 ± 10.7% for the CS-Str hydrogel). Reducing sugar analysis by DNS assay was used as an indirect method to determine lysozyme activity, since these reducing sugars are formed by the enzymatic cleavage of the glycosidic bond between two glucosamine-chitosan units and the subsequent release of chitosan fragments from the hydrogel into the supernatant.^42^ In this method, the functional aldehyde group of the reducing end of the polysaccharide is oxidized to a carboxyl group, and in the process, the yellow 3,5-dintrosalicylic acid compound is reduced to 3-amino, 5-nitrosalicylic acid, which has a reddish-brown color and can be detected by measuring the UV absorbance of the solution. Degradation of hydrogels was also studied using cryo SEM. As shown in Figure 3F, various pores form in the hydrogel scaffold during lysozyme-mediated degradation. On day 0, both hydrogels (CS and CS-Str) had comparable pore sizes and size distributions. On day 4, although the average pore sizes and size distributions of both hydrogels increased, the hydrogel CS exhibited a greater increase in pore size than the hydrogel CS-Str, which was attributed to the greater degradation of the former hydrogel due to the higher lytic activity of lysozyme against CS compared to that of CS-Str.

#### 3.2.2 Study of Hydrogels’ Antibiofilm Activity

The antibiofilm activity of CS hydrogels and CS-Str hydrogels was evaluated against pre-established (24 h) *E. coli* biofilms, first with gels directly loaded with a 10 mg/mL lysozyme solution so that the xerogels had a lysozyme concentration of about 700 μg/mg gel. To evaluate the antibiofilm activity of the hydrogels under more realistic conditions of dental disease caused by bacterial biofilms, nonloaded gels were exposed to artificial saliva^26,43,44^ containing lysozyme 10 μg/mL, instead of the lysozyme solution.

**Figure 4:**
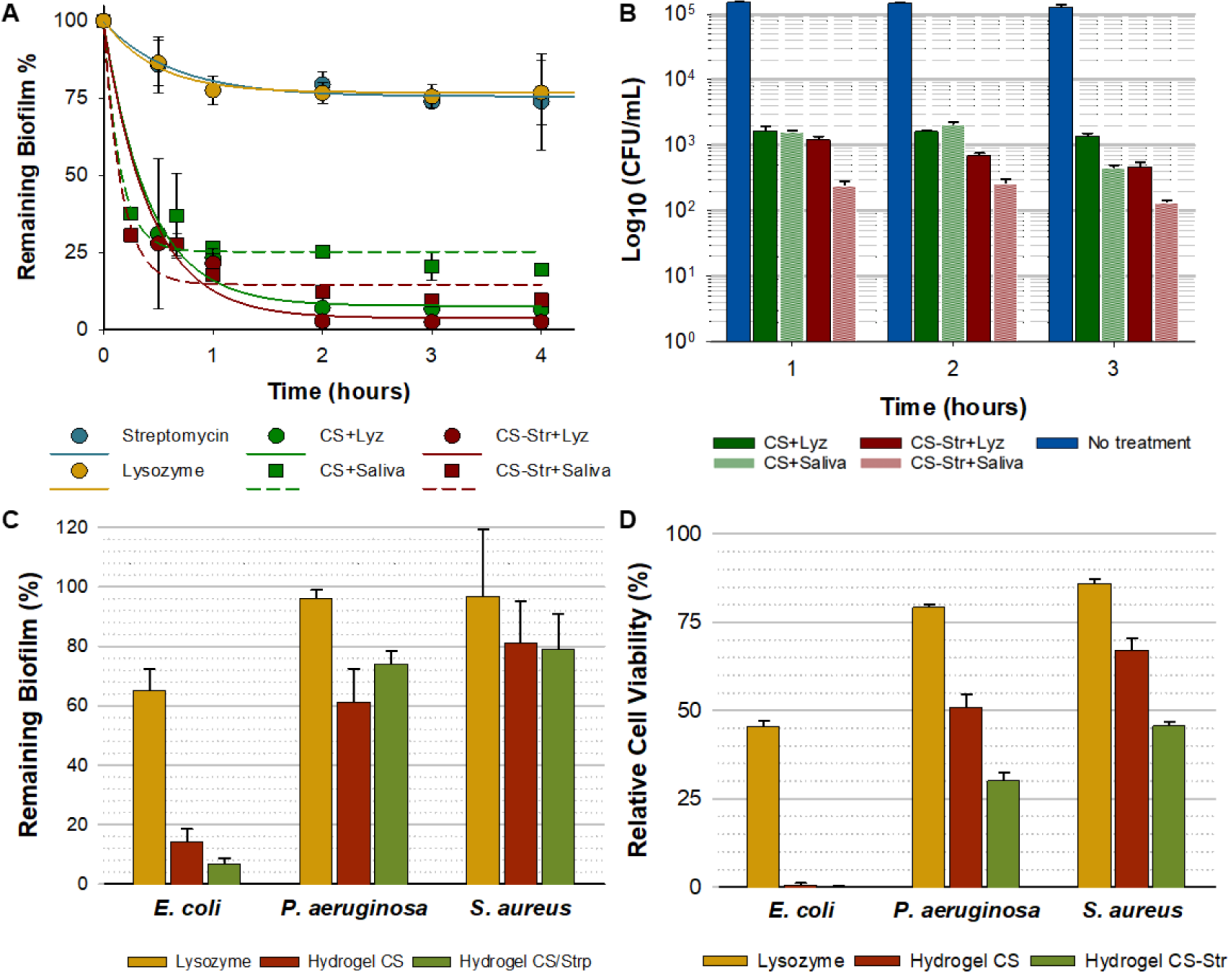
A) Loss of biomass from pre-established *E. coli* biofilms caused by CS and CS-Str hydrogels loaded with lysozyme or with artificial saliva. Results are expressed as percentages compared with those of untreated controls. B) Reduction of viable cells in *E. coli* biofilms after treatment with CS and CS-Str hydrogels, loaded with lysozyme or incubated with artificial saliva. Results are expressed as the log10 of the number of cells per mL obtained by CFU count ± SD. C) Biofilm biomass eradication on 24 h biofilms of *E. coli*, *P. aeruginosa*, and *S. aureus* preformed biofilms in the presence of 200 μL of artificial saliva containing 10 μg/mL of lysozyme after 24 h of treatment with CS and CS-Str hydrogels. D) Reduction of cell viability in *E. coli*, *P. aeruginosa*, and *S. aureus* biofilms after 3 h of treatment with CS and CS-Str hydrogels in the presence of 200 μL of artificial saliva containing 10 μg/mL of lysozyme. In C,D, results are expressed as percentages compared to untreated controls. In all cases, results represent the mean of three replicates. Error bars indicate standard deviations.

The results obtained show that both CS and CS-Str gels cause efficient eradication of *E. coli* biofilms, both from the point of view of biomass loss (Figure 4A) and the loss of bacterial viability within the biofilm (Figure 4B). Interestingly, the action of the chitosan gels in the eradication of the preformed biofilm is intense and relatively fast, as the maximum loss of biomass in the biofilm is reached in about 2 h in all cases (Figure 4A), with a loss of biomass of 75% to more than 95% eradication of the biofilm compared to the low 25% eradication that occurs when biofilms are treated with only a streptomycin or lysozyme solution.

The activity of the gels loaded with lysozyme differs slightly from that of the gels acting in the presence of artificial saliva, with the former being slightly higher. This difference can be explained by the greater amount of lysozyme present in the loaded gels, which implies a faster and more efficient degradation of chitosan, leading to a greater formation of COS, which, as we have seen previously (Figure 2), is ultimately responsible for the antibiofilm activity of chitosan hydrogels.

On the other hand, measurements of total biomass show no significant differences between the behavior of CS-Str hydrogels and that of CS. Therefore, quantification of viable cells in the biofilm was performed (Figure 4B). Viable cells in the biofilms were determined after treatment with the hydrogels by determining colony forming units (CFU) using LB-agar, which is still considered the gold standard among bacterial quantification methods, including biofilms.^45^ Although this method is time- and resource- consuming, it was chosen because it is known that numerous bacterial cells within a mature biofilm enter a latent stage in which their metabolic activity is almost or completely absent.^46^ The MTT or resazurin methods are unable to detect these dormant cells, but they are still viable and can resume their growth and multiplication when the conditions are more favorable. The results of the quantification of viable cells in the biofilm confirmed the different behavior of gels that contained streptomycin and gels that did not (Figure 4B). In the first case, when the hydrogel is hydrolyzed by lysozyme, chitosan-streptomycin conjugates are released in the biofilm, which can exert an antibiotic effect and gradually reduce the load of viable bacteria in the biofilm. Significantly, chitosan hydrogels that did not contain streptomycin also significantly reduced the population of viable bacteria within the biofilm compared to untreated biofilms. However, this reduction in bacterial viability was less than for CS-Str hydrogels and almost independent of exposure time (Figure 4B). This indicates that streptomycin retains its antibiotic activity even when bound to chitosan oligosaccharides, thereby enhancing the antibiotic effect already present in chitosan oligosaccharides.

Having thoroughly analyzed and verified the efficacy of CS and CS-Str hydrogels for the eradication of *E. coli* biofilms, we wanted to see if these hydrogels were also an effective means of combating more stubborn biofilms such as *Pseudomonas aeruginosa* and *Staphylococcus aureus*. To this end, we conducted preliminary studies on the ability of our hydrogels to eradicate preformed biofilms of these bacteria (Figures 4C,D). The comparative study was carried out only with the treatment of hydrogels in the presence of artificial saliva containing 10 μg/mL of lysozyme. As can be seen in Figure 4C, both CS and CS-Str hydrogels were able to reduce the biomass of *P. aeruginosa* and *S. aureus* biofilms, although with less intensity than in the case of *E. coli* biofilms. After 24 h of treatment, the biomass of *P. aeruginosa* biofilms was significantly reduced by around 40% with CS gels, whereas this reduction was lower, approximately 25%, with CS-Str hydrogels. The biomass of *S. aureus* biofilms was reduced by 20% in both cases. (Figure 4C). Similarly, the bacterial viability within the biofilm (Figure 4D) was also reduced. The bacterial viability, measured after 3 h of treatment with the gels, confirmed that the antibiotic streptomycin in the CS hydrogels had a significant cumulative effect on reducing the number of live bacteria. The antibiotic enhanced the reduction in bacterial viability within the biofilm by about 21% (Figure 4D). The assayed hydrogels affected cell viability more in *E. coli* biofilms than in *P. aeruginosa* and *S. aureus* biofilms. Among these two biofilms, the effect was significantly stronger in *P. aeruginosa* biofilms, where cell viability decreased by 48% and 70% with the CS and CS-Str hydrogels, respectively, than in *S. aureus* biofilms, where the CS-Str hydrogels caused the highest reduction in cell viability of 54% (Figure 4D). These differences could be due to the different degrees of disruption caused in the biofilms, which would result in better or worse accessibility of the antibiotic.

Interestingly, in our studies, chitosan hydrogels have shown enhanced antibiofilm activity against Gram– bacteria such as *E. coli* and *P. aeruginosa* than against biofilms of a Gram+ bacterium such as *S. aureus*.

### 3.3 *In Situ* Forming Chitosan and Chitosan-Streptomycin Hydrogels Serve as Platforms with Finely Tuned Degradability, Enabling On-Demand Release of Biofilm-Disrupting Enzymes and Efficient Eradication of Biofilms

#### 3.3.1 Preparation and Characterization of β-Glycerophosphate/Genipin Chitosan Hydrogels

The Gp-cross-linked chitosan hydrogels described above have demonstrated their potential applicability for the treatment of preformed biofilms in oral infections. However, these preformed hydrogels are difficult to deploy in the damage zone, which significantly limits their application in the dental field as implantable biomaterials.^47^ To overcome this drawback, we investigated the combination of physical cross-linking of chitosan by β-glycerophosphate (G2P) with covalent cross-linking by Gp to develop *in situ* forming hydrogels.^24^ A range of G2P concentrations (1.25, 1.87, and 2.5 M) was selected to analyze their influence on various properties such as gelation time, water absorption capacity, *in vitro* degradation, and antibiofilm activity.

**Figure 5:**
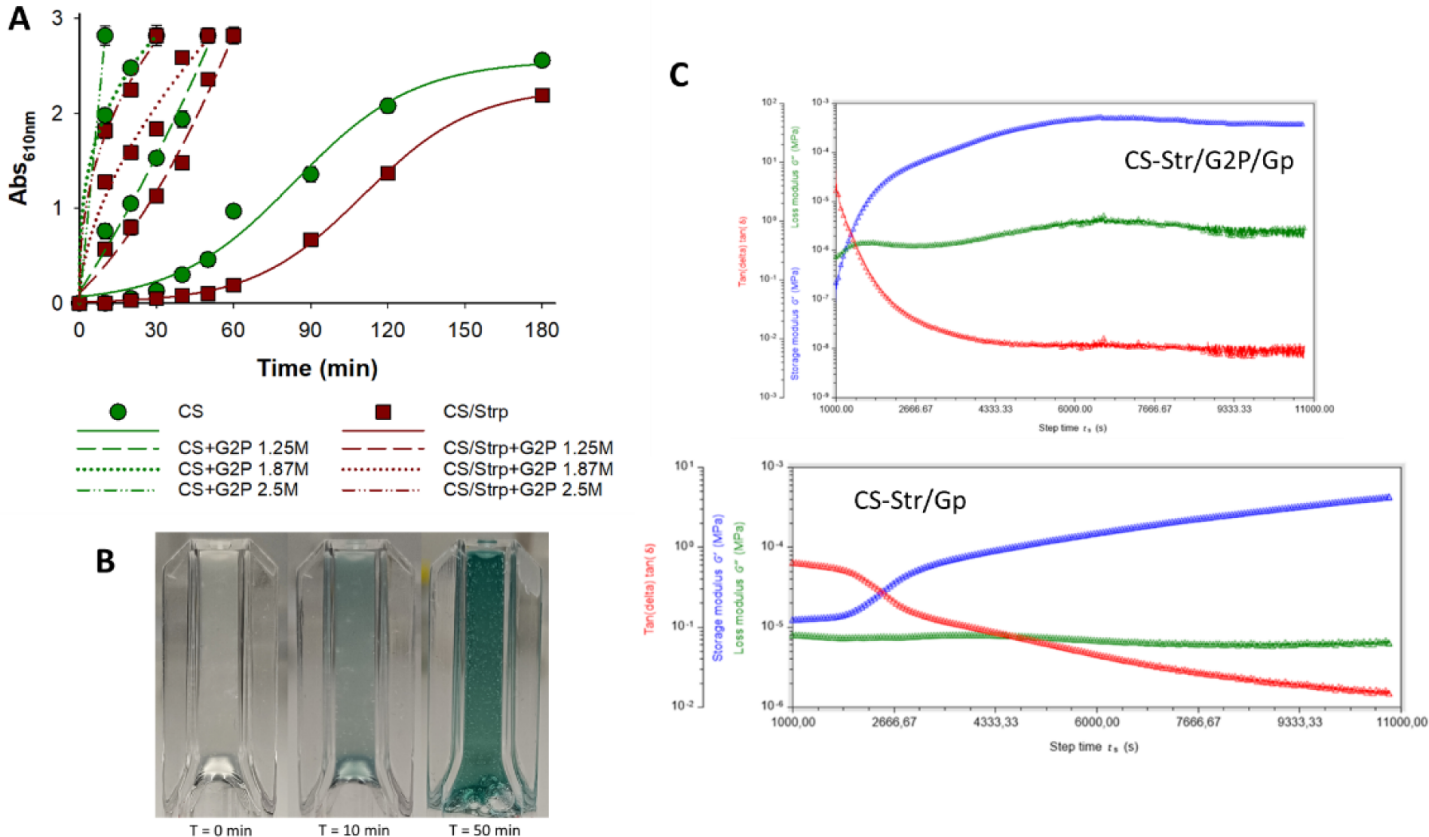
Synthesis and characterization of CS/G2P/Gp and CS-Str/G2P/Gp hydrogels. A) Absorbance evolution of CS and CS-Str hydrogels cross-linked with Gp and different concentrations of G2P (1.25 M, 1.87 M, or 2.5 M) at a wavelength of 610 nm. B) Macroscopic observation of the hydrogels over time (0, 10, and 50 min). C) Storage modulus (*G*′) (blue), loss modulus (*G*″) (green), and loss factor (tan (δ) = *G*″/*G*′) (red) versus time at 37 °C of CS-Str/G2P/Gp (1.87M) (top) and CS-Str/Gp (botton).

Absorbance spectral measurements at 610 nm, a well-documented absorption peak in the literature that is a characteristic of the Gp-CS bridge,^48^ were used to monitor the cross-linking rate at different times. As can be seen in Figure 5A, CS/G2P/Gp or CS-Str/G2P/Gp hydrogels have a shorter gelation time than chemically cross-linked gels (CS/Gp or CS-Str/Gp), as the kinetics of the two gelation types differ significantly as previously reported.^24^ For these hydrogels, the observed absorbance (data not shown) after 24 h of gelation is very similar to that after 12 h.

This could indicate that at this absorbance (2.82), all cross-links in the gel network have been formed. When G2P is added, this absorbance value is reached much earlier, indicating an interesting reduction in gelation time, with the fastest gelation occurring in 10 min when a G2P concentration of 2.5 M was used.

The effect of different gelation times on the visual appearance of CS/G2P/Gp (1.87M) hydrogels is shown in Figure 5B. At 37 °C, the color of the hydrogel changes from colorless (*t* = 0 min) to a green shade (*t* = 10 min) and finally to a dark blue coloration (*t* = 50 min) with increasing gelation time. These observations can be explained based on the fact that, in the presence of G2P, CS solutions at 37 °C turn into a colorless hydrogel instantly due to the ionic interaction between chitosan amine groups and negative phosphate groups of G2P. However, at longer times, the hydrogel turns blue, indicating the covalent linkage of Gp to chitosan free amine moieties. The gelling process was then investigated in time-sweep experiments by monitoring the changes in the shear moduli over time using rheological experiments.^49^Figure 5C shows the values of the storage modulus *G*′ and the loss *G″* modulus, which represent the solid-like and liquid-like characteristics, respectively, as well as the loss or damping factor tan (δ) (*G″*/*G*′), a factor that indicates the ratio between the elastic part and the viscous part. The decay of tan (δ) provides information about the gelation process, because the greater the difference between loss and storage modulus (*G″* or *G*′), the closer the value of tan (δ) is to 0.^49^ The comparison of the slopes of tan (δ) in Figure 5C shows that the decay during gelation of CS-Str and Gp is more abrupt in the presence of G2P than in the absence of this additive. While tan (δ) in the CS-Str/G2P/Gp hydrogel reached the horizontal asymptote after 80 min (4800 s) of the experiment, tan (δ) in CS-Str/Gp did not reach this asymptote throughout the experiment, indicating a faster gelation rate for the CS-Str/G2P/Gp hydrogel.

**Figure 6:**
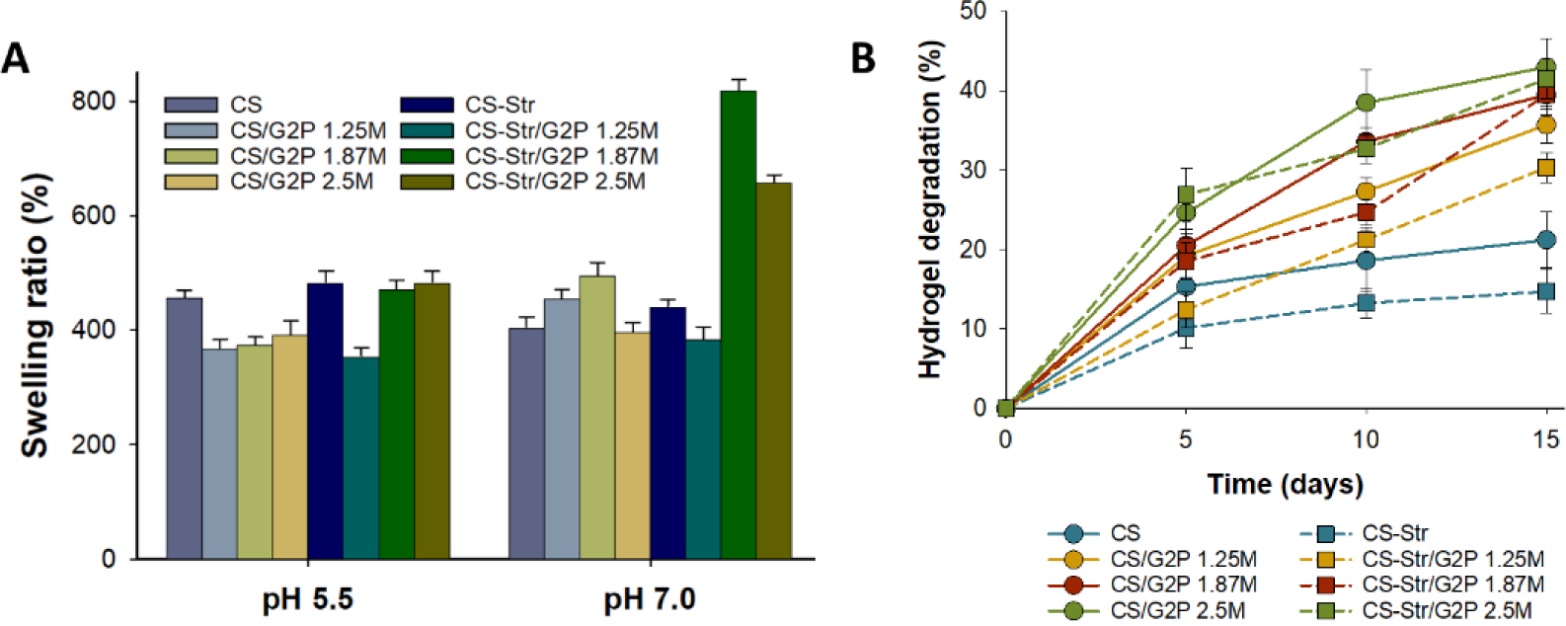
Characterization of the CS/G2P/Gp and CS-Str/G2P/Gp hydrogels. A) Swelling behavior of CS- and CS-Str-based hydrogels at pH 5.5 and 7.0. B) Degradation of hydrogels after 5, 10, and 15 days. In both cases, the values are the average of three replicates, and the standard deviation is given.

Figure 6A shows that the hydrogels formed with different G2P concentrations differ not only in their gelling time but also in their swelling capacity. While in the absence of G2P, the highest degrees of swelling were observed at a pH value of 5.5, a different behavior was observed when G2P was added. This could be due to the fact that in covalent cross-linking with genipin of cocrosslinked hydrogels, in contrast to pure covalent hydrogels, where cross-linking occurs at acidic pH, the homopolymerization of genipin is supported as a consequence of the nonacidic pH of the medium,^48^ especially at high concentrations of G2P.

Thus, genipin homopolymerization in cocrosslinked hydrogels leads to looser networks as a consequence of the larger segment chains between the covalent cross-linking points, which could explain the greater swelling capacity of cocrosslinked hydrogels than that expected after the summative effect of covalent cross-linking to form physical gels.^24^

Finally, the mass loss (%) of the hydrogels over time was determined as a measure of degradation (Figure 6B). Although a slow and continuous degradation behavior of hydrogels is observed, indicating a controlled release of CS or CS-Str oligosaccharides, it should be noted that the mass loss of the cocross-linking hydrogels in the saliva medium with Lyz (10 μg/mL) is higher than that of pure covalent hydrogels. While the percentage degradation values after 15 days for the CS/Gp and CS-Str/Gp hydrogels were 21.2 ± 3.5% and 14.7 ± 2.8%, the percentage degradation values for CS/G2P/Gp and CS-Str/G2P/Gp hydrogels were 35.7 ± 2.3%, 39.5 ± 2.6% and 43.0 ± 3.5% for CS-based hydrogels and 30.3 ± 1.9%, 39.5 ± 1.8%, and 41.5 ± 2.5% for CS-Str-based hydrogels. This is due to the fact that physical cross-linking of cocross-linked hydrogels tends to degrade faster than the covalent cross-linking.

#### 3.3.2 Citotoxicity

**Figure 7:**
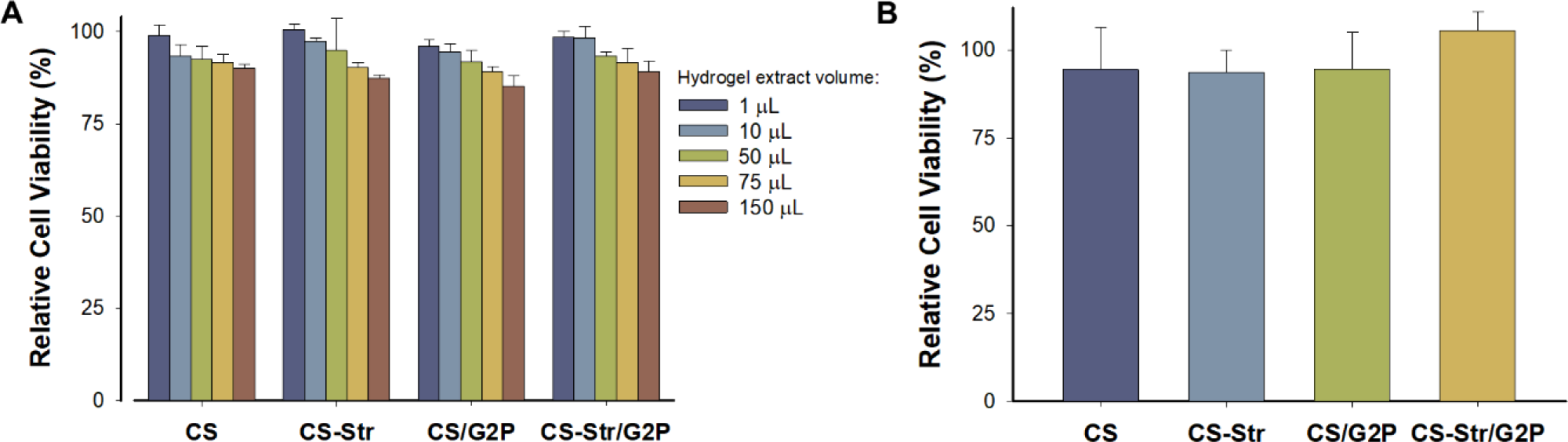
Assessment of hydrogel biocompatibility via Resazurin assay on A549 cells post 24-h treatment: A) Cytotoxicity evaluation via serial dilutions of hydrogel extracts derived from incubated hydrogel in saliva medium with Lyz (details in Experimental section); B) Cytotoxicity assessment using hydrogel fragments. Results represent the mean of three replicates. Error bars indicate standard deviations.

The biocompatibility of the prepared hydrogels was assessed using the Resazurin method^50^ on A549 cells, an epithelial cell line derived from lung adenocarcinoma^51^ widely employed in toxicology research.^52^ First, extracts from the hydrogels obtained via lysozyme digestion were evaluated for cytotoxicity (Figure 7A). Subsequently, the cytotoxicity of fragments of the hydrogels applied directly to the A549 cell cultures was tested (Figure 7B).

Both experiments yielded cell viabilities exceeding 85% across the tested concentrations, indicating no significant cytotoxic effects. In the first case, the extracts of the four types of gels prepared allowed cell viability of over 85% for each of the amounts tested and did not exhibit significant cytotoxicity. A similar result was obtained when fragments of the hydrogels were tested directly. These findings unequivocally demonstrate the noncytotoxic nature of the hydrogels toward A549 cells.

#### 3.3.3 Study of *In Situ* Forming Hydrogels Antibiofilm Activity

**Figure 8:**
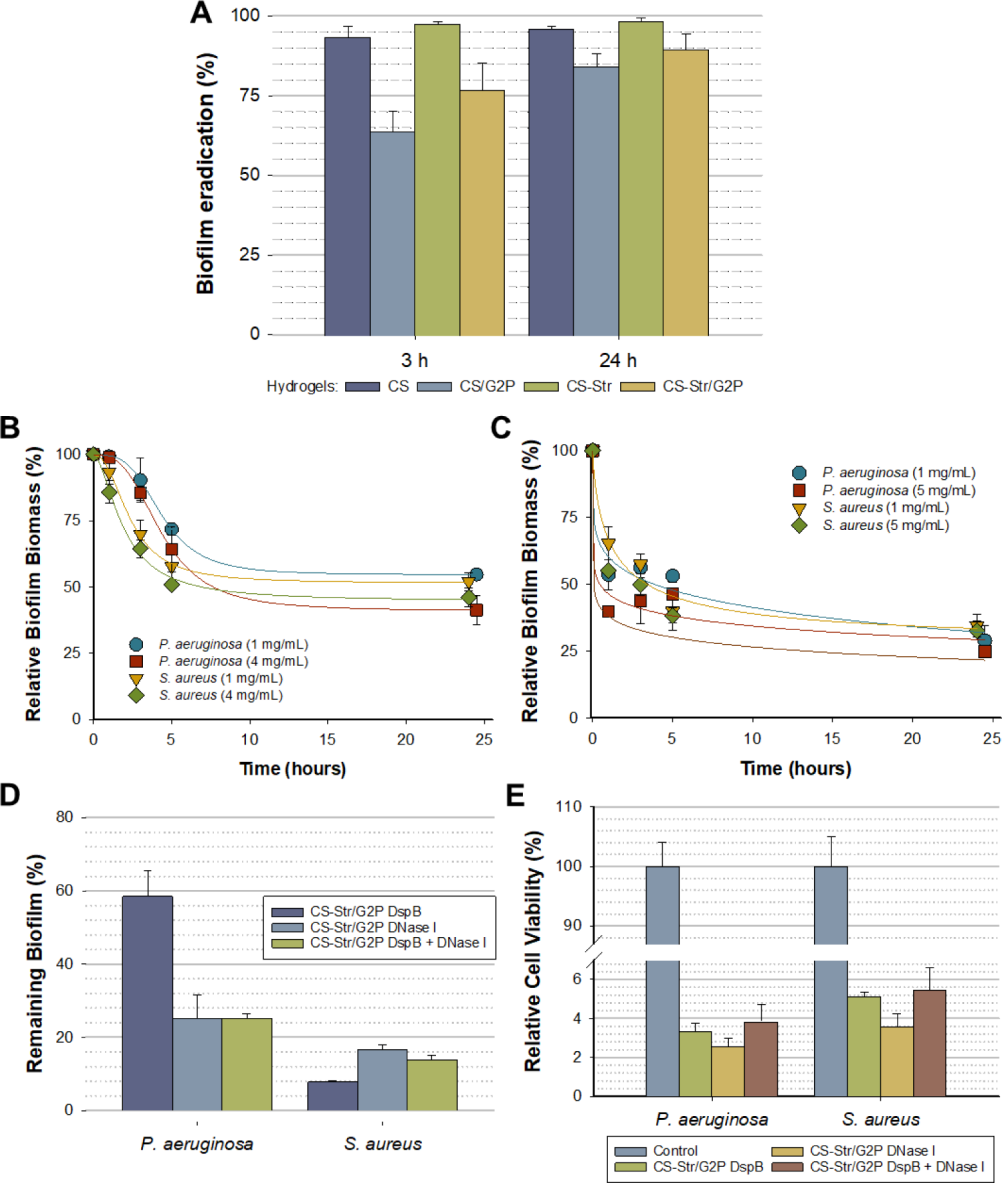
Analysis of the antibiofilm activity of *in situ* forming CS and CS-Str hydrogels: A) Comparison of the ability of CS and CS-Str hydrogels gelled with and without G2P to remove *E. coli* biofilms; B) and C) Eradication kinetics of *P. aeruginosa* and *S. aureus* biofilms with DspB (B) and DNase I (C); D) Biofilm biomass reduction after 24 h of treatment; E) Reduction of cell viability after 3 h of treatment, expressed as a percentage of colony-forming unit (CFU) with respect to a biofilm that has not been treated (control). Results represent the mean of three replicates. Error bars indicate standard deviations.

To determine whether the incorporation of G2P into the hydrogel altered its antibiofilm activity, we analyzed whether the ability to eradicate *E. coli* biofilms was comparable to that of hydrogels cross-linked with genipin alone. As can be seen in Figure 8A, the gels with G2P exhibit lower or slower antibiofilm activity, as at a relatively short treatment time of 3 h, biofilm eradication was 30% lower for the CS gels and about 20% lower for the CS-Str gels. After 24 h of treatment, the percentage of biofilm eradicated increased for both hydrogel types but without reaching the level of eradication achieved with genipin-only cross-linked hydrogels (Figure 8A).

From the results obtained with the *E. coli* biofilms, it can be assumed that *in situ* forming hydrogels will behave similarly with more resistant biofilms such as those of *P. aeruginosa* and *S. aureus*, i.e., they will have a lower capacity to eradicate these biofilms than the hydrogels cross-linked with genipin. To increase the ability of these hydrogels to eradicate biofilms, we decided to evaluate the use of biofilm-dispersing enzymes.

Enzymatic degradation of the biofilm matrix, involving glycosidases and deoxyribonucleases, is crucial for detaching cells from the biofilm colonies. These enzymes break down the biofilm matrix polysaccharides and DNA, allowing cells to detach and disperse and making them susceptible to antibiotics and the host immune system.^8,53^ In fact, both free DspB and DNase I are able to dissolve biofilms of *P. aeruginosa* and *S. aureus*. However, eradication occurs almost completely in the first 5 h of treatment, with no significant increase in biofilm biomass loss observed when treatment was extended beyond 24 h (Figure 8B,C). Considering that enzymatic stability is an aspect that needs to be taken into account when using enzymes to disperse biofilms, and that dispersing enzymes *per se* do not have a bactericidal effect,^8^ we decided to encapsulate these enzymes in our CS-Str hydrogels, creating a multifunctional material, capable of both dispersing the biofilm and eliminating the cells within it, thus avoiding the possibility of the dispersed cells colonizing new areas and leading to expansion of the infection or new infections.

CS and CS-Str hydrogels containing disrupting enzymes were prepared as described previously (section 3.3.1), adding to the mixture 5 μL of 1 mg/mL or/and 100 μg/mL of DspB and DNase I, respectively. As can be seen in Figure 8D, the biomass of *P. aeruginosa* and *S. aureus* biofilms was significantly reduced after treatment with the hydrogels containing disrupting enzymes, and in all cases, this reduction was greater than with the gels that did not contain dispersing enzymes (Figure 5). A differential effect can be observed for the biofilms of *P. aeruginosa* and *S. aureus*. The eradication of the biofilm is significantly greater in the case of *S. aureus*, whereby the biomass of the biofilms was reduced by between 84% with DNase I and up to 92% in the case of DspB. This result is consistent with the fact that one of the main components of the extracellular matrix of *S. aureus* biofilms is a polysaccharide composed of β-1,6-linked *N*-acetylglucosamine.^54^ The combination of the two dispersing enzymes showed no additive effect, although the reduction in biofilm biomass was 4% greater than that when DNase I was used alone (Figure 8D). As previously mentioned, the behavior of hydrogels loaded with disruptive enzymes was different in the case of *P. aeruginosa* biofilms. In this case, the effect of DspB was significantly lower, and a reduction in biofilm biomass of only 43% was achieved. The reduction in biofilm biomass caused by DNase I was much greater, reaching 73%. Also in this case, no cumulative effect was observed when the two enzymes, DspB and DNase I, were combined in a single gel, so that the observed biomass reduction was practically identical with that obtained with DNase I alone. The effect of decomposition of EPS on facilitating access of the antibiotic to the cells can be clearly observed in Figure 8E. In all cases, the reduction in cell viability was equal to or greater than 95%. A small difference can be observed in the sensitivity to the antibiotic, which is slightly higher in the case of *P. aeruginosa*. In all cases, the reduction in cell viability was significantly greater when disruptive enzymes were used than when gels without enzymes were used (Figure 5B). The cell viability decreased from about 30% to less than 4% in the case of *P. aeruginosa* biofilms and from 45% to 5% in the case of *S. aureus* biofilms.

## 4 Conclusion

In conclusion, this study presents the development of an *in situ* forming chitosan-streptomycin hydrogel (CS-Str) to be used for the treatment of oral infections, such as periodontitis and caries, by targeting established bacterial biofilms. The hydrogel reacts to lysozyme, an enzyme overexpressed in saliva during oral infections, by breaking down and releasing chitosan-streptomycin oligosaccharides that eliminate the biofilms.

The CS-Str hydrogel shows significant *in vitro* efficacy against biofilms of *E. coli*, *S. aureus*, and *P. aeruginosa* by reducing both the biomass of the biofilm and viable bacterial populations. Notably, the hydrogel exhibits synergistic effects between chitosan and streptomycin that enhance its antibiofilm activity.

In addition, the incorporation of biofilm-disrupting enzymes such as DNase I and DspB into the hydrogel significantly enhances its ability to reduce the biofilm biomass of *P. aeruginosa* and *S. aureus* by over 84% and up to 92%, respectively. This enzymatic load leads to a drastic reduction in cell viability, with levels falling below 4% for *P. aeruginosa* and below 5% for *S. aureus*.

Overall, the CS-Str hydrogel represents a promising biomaterial for the treatment of oral infections, providing controlled degradation and sustained antibiofilm activity, thus advancing therapeutic strategies to combat persistent biofilms.

## Supporting Information Available

Preliminary assays for chitosan selection, experimental protocols for determination of chitosan acetylation degree, experimental protocols for measurement of lysozyme activity by determination of reducing sugars using the Park Johnson assay, and ^1^H NMR spectra for CS-Str and representative photographs of plate bacterial count of hydrogels and biofilm for *P. aeruginosa* and *S. aureus* (PDF)

## Abbreviations

CS: chitosan
CS-Str: chitosan-streptomycin conjugates
DA: degree of acetylation
CD: cross-linking degree
EPS: extracellular polymeric substances
DspB: Dispersin B
Lyz: lysozyme
Gp: genipin
G2P: β-glycerophosphate
LVER: linear viscoelastic region
CFU: colony forming units
MTT: 3-(4,5-dimethylthiazol-2-yl)-2,5-diphenyltetrazolium bromide

## References

[ref1] Rosan B.; Lamont R. J. Dental Plaque Formation. Microbes. Infect. 2000, 2 (13), 1599–1607. 10.1016/S1286-4579(00)01316-2. 11113379

[ref2] Fernandes T.; Bhavsar C.; Sawarkar S.; D’souza A. Current and Novel Approaches for Control of Dental Biofilm. Int. J. Pharm. 2018, 536 (1), 199–210. 10.1016/j.ijpharm.2017.11.019. 29157962

[ref3] Martínez-Hernández M.; Reda B.; Hannig M. Chlorhexidine Rinsing Inhibits Biofilm Formation and Causes Biofilm Disruption on Dental Enamel in Situ. Clin. Oral Investig. 2020, 24 (11), 3843–3853. 10.1007/s00784-020-03250-3. 32125530

[ref4] Boucher H. W.; Talbot G. H.; Bradley J. S.; Edwards J. E.; Gilbert D.; Rice L. B.; Scheld M.; Spellberg B.; Bartlett J. Bad Bugs, No Drugs: No ESKAPE! An Update from the Infectious Diseases Society of America. Clin. Infect. Dis. 2009, 48 (1), 1–12. 10.1086/595011. 19035777

[ref5] Spellberg B.; Powers J. H.; Brass E. P.; Miller L. G.; Edwards J. E. Jr. Trends in Antimicrobial Drug Development: Implications for the Future. Clin. Infect. Dis. 2004, 38 (9), 1279–1286. 10.1086/420937. 15127341

[ref6] McCoy L. C.; Wehler C. J.; Rich S. E.; Garcia R. I.; Miller D. R.; Jones J. A. Adverse Events Associated with Chlorhexidine Use: Results from the Department of Veterans Affairs Dental Diabetes Study. J. Am. Dent. Assoc. 2008, 139 (2), 178–183. 10.14219/jada.archive.2008.0134. 18245686

[ref7] Azeredo J.; García P.; Drulis-Kawa Z. Targeting Biofilms Using Phages and Their Enzymes. Curr. Opin. Biotechnol. 2021, 68, 251–261. 10.1016/j.copbio.2021.02.002. 33714050

[ref8] Wang S.; Zhao Y.; Breslawec A. P.; Liang T.; Deng Z.; Kuperman L. L.; Yu Q. Strategy to Combat Biofilms: A Focus on Biofilm Dispersal Enzymes. Biofilms Microbiomes 2023, 9 (1), 63–77. 10.1038/s41522-023-00427-y. 37679355 PMC10485009

[ref9] Yari M.; Ghoshoon M. B.; Vakili B.; Ghasemi Y. Therapeutic Enzymes: Applications and Approaches to Pharmacological Improvement. Curr. Pharm. Biotechnol. 2017, 18 (7), 531–540. 10.2174/1389201018666170808150742. 28786356

[ref10] Luo H.; Yin X.-Q.; Tan P.-F.; Gu Z.-P.; Liu Z.-M.; Tan L. Polymeric Antibacterial Materials: Design, Platforms and Applications. J. Mater. Chem. B 2021, 9 (12), 2802–2815. 10.1039/D1TB00109D. 33710247

[ref11] Tiburcio E.; García-Junceda E.; Garrido L.; Fernández-Mayoralas A.; Revuelta J.; Bastida A. Preparation and Characterization of Aminoglycoside-Loaded Chitosan/Tripolyphosphate/Alginate Microspheres against *E. Coli*. Polymers 2021, 13 (19), 3326 10.3390/polym13193326. 34641142 PMC8512199

[ref12] Thottathil S.; Puttaiahgowda Y. M.; Kanth S. Advancement and Future Perspectives on Ampicillin-Loaded Antimicrobial Polymers- A Review. J. Drug Delivery Sci. Technol. 2023, 81, 104227 10.1016/j.jddst.2023.104227.

[ref13] Thakor P.; Bhavana V.; Sharma R.; Srivastava S.; Singh S. B.; Mehra N. K. Polymer–Drug Conjugates: Recent Advances and Future Perspectives. Drug Discovery Today 2020, 25 (9), 1718–1726. 10.1016/j.drudis.2020.06.028. 32629170

[ref14] Junceda-Mena I.; García-Junceda E.; Revuelta J. From the Problem to the Solution: Chitosan Valorization Cycle. Carbohydr. Polym. 2023, 309, 120674 10.1016/j.carbpol.2023.120674. 36906370

[ref15] Orgaz B.; Lobete M. M.; Puga C. H.; San Jose C. Effectiveness of Chitosan against Mature Biofilms Formed by Food Related Bacteria. Int. J. Mol. Sci. 2011, 12 (1), 817–828. 10.3390/ijms12010817. 21340015 PMC3039981

[ref16] Cicciù M.; Fiorillo L.; Cervino G. Chitosan Use in Dentistry: A Systematic Review of Recent Clinical Studies. Mar. Drugs 2019, 17 (7), 417–432. 10.3390/md17070417. 31319609 PMC6669505

[ref17] Revuelta J.; Fraile I.; Monterrey D. T.; Peña N.; Benito-Arenas R.; Bastida A.; Fernández-Mayoralas A.; García-Junceda E. Heparanized Chitosans: Towards the Third Generation of Chitinous Biomaterials. Mater. Horiz. 2021, 8 (10), 2596–2614. 10.1039/D1MH00728A. 34617543

[ref18] Revuelta J.; Aranaz I.; Acosta N.; Civera C.; Bastida A.; Peña N.; Monterrey D. T.; Doncel-Pérez E.; Garrido L.; Heras Á.; García-Junceda E.; Fernández-Mayoralas A. Unraveling the Structural Landscape of Chitosan-Based Heparan Sulfate Mimics Binding to Growth Factors: Deciphering Structural Determinants for Optimal Activity. ACS Appl. Mater. Interfaces 2020, 12 (23), 25534–25545. 10.1021/acsami.0c03074. 32426965

[ref19] Aguanell A.; Del Pozo M. L.; Pérez-Martín C.; Pontes G.; Bastida A.; Fernández-Mayoralas A.; García-Junceda E.; Revuelta J. Chitosan Sulfate-Lysozyme Hybrid Hydrogels as Platforms with Fine-Tuned Degradability and Sustained Inherent Antibiotic and Antioxidant Activities. Carbohydr. Polym. 2022, 291, 119611 10.1016/j.carbpol.2022.119611. 35698348

[ref20] Zhang A.; Mu H.; Zhang W.; Cui G.; Zhu J.; Duan J. Chitosan Coupling Makes Microbial Biofilms Susceptible to Antibiotics. Sci. Rep. 2013, 3 (1), 3364 10.1038/srep03364. 24284335 PMC3842539

[ref21] Tré-Hardy M.; Vanderbist F.; Traore H.; Devleeschouwer M. J. In Vitro Activity of Antibiotic Combinations against Pseudomonas Aeruginosa Biofilm and Planktonic Cultures. Int. J. Antimicrob. Agents 2008, 31 (4), 329–336. 10.1016/j.ijantimicag.2007.12.005. 18280117

[ref22] Li R.; Yuan X.; Wei J.; Zhang X.; Cheng G.; Wang Z.; Du Y. Synthesis and Evaluation of a Chitosan Oligosaccharide-Streptomycin Conjugate against Pseudomonas Aeruginosa Biofilms. Mar. Drugs 2019, 17 (1), 43 10.3390/md17010043. 30634609 PMC6356912

[ref23] Heimbuck A. M.; Priddy-Arrington T. R.; Padgett M. L.; Llamas C. B.; Barnett H. H.; Bunnell B. A.; Caldorera-Moore M. E. Development of Responsive Chitosan–Genipin Hydrogels for the Treatment of Wounds. ACS Appl. Bio Mater. 2019, 2 (7), 2879–2888. 10.1021/acsabm.9b00266. 35030822

[ref24] Maiz-Fernández S.; Guaresti O.; Pérez-Álvarez L.; Ruiz-Rubio L.; Gabilondo N.; Vilas-Vilela J. L.; Lanceros-Mendez S. β-Glycerol Phosphate/Genipin Chitosan Hydrogels: A Comparative Study of Their Properties and Diclofenac Delivery. Carbohydr. Polym. 2020, 248, 116811 10.1016/j.carbpol.2020.116811. 32919543

[ref25] Fraile-Gutiérrez I.; Iglesias S.; Acosta N.; Revuelta J. Chitosan-Based Oral Hydrogel Formulations of β-Galactosidase to Improve Enzyme Supplementation Therapy for Lactose Intolerance. Int. J. Biol. Macromol. 2024, 255, 127755 10.1016/j.ijbiomac.2023.127755. 37935291

[ref26] Tonguc Altin K.; Topcuoglu N.; Duman G.; Unsal M.; Celik A.; Selvi Kuvvetli S.; Kasikci E.; Sahin F.; Kulekci G. Antibacterial Effects of Saliva Substitutes Containing Lysozyme or Lactoferrin against Streptococcus Mutans. Arch. Oral Biol. 2021, 129, 105183 10.1016/j.archoralbio.2021.105183. 34091207

[ref27] Danese P. N.; Pratt L. A.; Dove S. L.; Kolter R. The Outer Membrane Protein, Antigen 43, Mediates Cell-to-Cell Interactions within *Escherichia Coli* Biofilms. Mol. Microbiol. 2000, 37 (2), 424–432. 10.1046/j.1365-2958.2000.02008.x. 10931336

[ref28] Pezzoni M.; Pizarro R.; Costa C. Evaluation of Viable Cells in Pseudomonas Aeruginosa Biofilms by Colony Count and Live/Dead Staining. BIO-Protocol 2020, 10 (18), e3762 10.21769/BioProtoc.3762. 33659420 PMC7842345

[ref29] Yap C. H.; Ramle A. Q.; Lim S. K.; Rames A.; Tay S. T.; Chin S. P.; Kiew L. V.; Tiekink E. R. T.; Chee C. F. Synthesis and Staphylococcus Aureus Biofilm Inhibitory Activity of Indolenine-Substituted Pyrazole and Pyrimido[1,2-b]Indazole Derivatives. Bioorg. Med. Chem. 2023, 95, 117485 10.1016/j.bmc.2023.117485. 37812886

[ref30] Kean T.; Thanou M. Biodegradation, biodistribution and toxicity of chitosan. Adv. Drug Delivery Rev. 2010, 62 (1), 3–11. 10.1016/j.addr.2009.09.004. 19800377

[ref31] Lončarević A.; Ivanković M.; Rogina A. Lysozyme-Induced Degradation of Chitosan: The Characterisation of Degraded Chitosan Scaffolds. J. Tissue Repair Regen. 2017, 1 (1), 12–22. 10.14302/issn.2640-6403.jtrr-17-1840.

[ref32] Vårum K. M.; Myhr M. M.; Hjerde R. J. N.; Smidsrød O. In Vitro Degradation Rates of Partially N-Acetylated Chitosans in Human Serum. Carbohydr. Res. 1997, 299 (1), 99–101. 10.1016/S0008-6215(96)00332-1. 9129298

[ref33] Bagheri-Khoulenjani S.; Taghizadeh S. M.; Mirzadeh H. An Investigation on the Short-Term Biodegradability of Chitosan with Various Molecular Weights and Degrees of Deacetylation. Carbohydr. Polym. 2009, 78 (4), 773–778. 10.1016/j.carbpol.2009.06.020.

[ref34] Thompson J. S.; Shockman G. D. A Modification of the Park and Johnson Reducing Sugar Determination Suitable for the Assay of Insoluble Materials: Its Application to Bacterial Cell Walls. Anal. Biochem. 1968, 22 (2), 260–268. 10.1016/0003-2697(68)90315-1. 4966716

[ref35] Chávez de Paz L. E.; Resin A.; Howard K. A.; Sutherland D. S.; Wejse P. L. Antimicrobial Effect of Chitosan Nanoparticles on Streptococcus Mutans Biofilms. Appl. Environ. Microbiol. 2011, 77 (11), 3892–3895. 10.1128/AEM.02941-10. 21498764 PMC3127608

[ref36] Rabea E. I.; Badawy M. E.-T.; Stevens C. V.; Smagghe G.; Steurbaut W. Chitosan as Antimicrobial Agent: Applications and Mode of Action. Biomacromolecules 2003, 4 (6), 1457–1465. 10.1021/bm034130m. 14606868

[ref37] Merritt J. H.; Kadouri D. E.; O’Toole G. A. Growing and Analyzing Static Biofilms. Curr. Protoc. Microbiol. 2005, Chapter 1 (1), Unit 1B.1 10.1002/9780471729259.mc01b01s22. PMC4568995 18770545

[ref38] Fu J.; Yang F.; Guo Z. The Chitosan Hydrogels: From Structure to Function. New J. Chem. 2018, 42 (21), 17162–17180. 10.1039/C8NJ03482F.

[ref39] Muzzarelli R. A. A. Genipin-Crosslinked Chitosan Hydrogels as Biomedical and Pharmaceutical Aids. Carbohydr. Polym. 2009, 77 (1), 1–9. 10.1016/j.carbpol.2009.01.016.

[ref40] Sung H.-W.; Huang R.-N.; Huang L. L. H.; Tsai C.-C. In Vitro Evaluation of Cytotoxicity of a Naturally Occurring Cross-Linking Reagent for Biological Tissue Fixation. J. Biomater. Sci., Polym. Ed. 1999, 10 (1), 63–78. 10.1163/156856299X00289. 10091923

[ref41] Jin J.; Song M.; Hourston D. J. Novel Chitosan-Based Films Cross-Linked by Genipin with Improved Physical Properties. Biomacromolecules 2004, 5 (1), 162–168. 10.1021/bm034286m. 14715022

[ref42] McKee L. S. Measuring Enzyme Kinetics of Glycoside Hydrolases Using the 3,5-Dinitrosalicylic Acid Assay BT - Protein-Carbohydrate Interactions: methods and Protocols; Abbott D. W.; Lammerts van Bueren A., Eds.; Springer New York: New York, NY, 2017; pp. 27 36. DOI: 10.1007/978-1-4939-6899-2_3. 28417358

[ref43] Leung V. W.-H.; Darvell B. W. Artificial Salivas for in Vitro Studies of Dental Materials. J. Dent. 1997, 25 (6), 475–484. 10.1016/S0300-5712(96)00068-1. 9604578

[ref44] Gal J. About a Synthetic Saliva for in Vitro Studies. Talanta 2001, 53 (6), 1103–1115. 10.1016/S0039-9140(00)00618-4. 18968202

[ref45] Azeredo J.; Azevedo N. F.; Briandet R.; Cerca N.; Coenye T.; Costa A. R.; Desvaux M.; Di Bonaventura G.; Hébraud M.; Jaglic Z.; Kačániová M.; Knøchel S.; Lourenço A.; Mergulhão F.; Meyer R. L.; Nychas G.; Simões M.; Tresse O.; Sternberg C. Critical Review on Biofilm Methods. Crit. Rev. Microbiol. 2017, 43 (3), 313–351. 10.1080/1040841X.2016.1208146. 27868469

[ref46] Fedorowicz J.; Cruz C. D.; Morawska M.; Ciura K.; Gilbert-Girard S.; Mazur L.; Mäkkylä H.; Ilina P.; Savijoki K.; Fallarero A.; Tammela P.; Sączewski J. Antibacterial and Antibiofilm Activity of Permanently Ionized Quaternary Ammonium Fluoroquinolones. Eur. J. Med. Chem. 2023, 254, 115373 10.1016/j.ejmech.2023.115373. 37084595

[ref47] Lee J. H. Injectable Hydrogels Delivering Therapeutic Agents for Disease Treatment and Tissue Engineering. Biomater. Res. 2018, 22 (1), 27 10.1186/s40824-018-0138-6. 30275970 PMC6158836

[ref48] Mi F.-L.; Shyu S.-S.; Peng C.-K. Characterization of Ring-Opening Polymerization of Genipin and PH-Dependent Cross-Linking Reactions between Chitosan and Genipin. J. Polym. Sci., Part A: Polym. Chem. 2005, 43 (10), 1985–2000. 10.1002/pola.20669.

[ref49] Moura M. J.; Figueiredo M. M.; Gil M. H. Rheological Study of Genipin Cross-Linked Chitosan Hydrogels. Biomacromolecules 2007, 8 (12), 3823–3829. 10.1021/bm700762w. 18004810

[ref50] Rodríguez-Corrales J. A.; Josan J. S. Resazurin Live Cell Assay: Setup and Fine-Tuning for Reliable Cytotoxicity Results. In Proteomics for Drug Discovery. Methods in Molecular Biology, Lazar I.; Kontoyianni M.; Lazar A., Eds.; Humana Press: New York, NY, 2017; pp. 207–219. 10.1007/978-1-4939-7201-2_14 28809005

[ref51] Foster K. A.; Oster C. G.; Mayer M. M.; Avery M. L.; Audus K. L. Characterization of the A549 Cell Line as a Type II Pulmonary Epithelial Cell Model for Drug Metabolism. Exp. Cell Res. 1998, 243, 359–366. 10.1006/excr.1998.4172. 9743595

[ref52] O’Loughlin C. T.; Miller L. C.; Siryaporn A.; Drescher K.; Semmelhack M. F.; Bassler B. L. A Quorum-Sensing Inhibitor Blocks Pseudomonas Aeruginosa Virulence and Biofilm Formation. Proc. Natl. Acad. Sci. U. S. A. 2013, 110 (44), 17981–17986. 10.1073/pnas.1316981110. 24143808 PMC3816427

[ref53] Kaplan J. B. Biofilm Dispersal: Mechanisms, Clinical Implications, and Potential Therapeutic Uses. J. Dent. Res. 2010, 89 (3), 205–218. 10.1177/0022034509359403. 20139339 PMC3318030

[ref54] Lister J. L.; Horswill A. R. *Staphylococcus aureus* Biofilms: Recent Developments in Biofilm Dispersal. Front. Cell. Infect. Microbiol. 2014, 4, 178 10.3389/fcimb.2014.00178. 25566513 PMC4275032

