# Supplementary material for: Lysozyme-Responsive Hydrogels of Chitosan-Streptomycin Conjugates for the On-Demand Release of Biofilm-Dispersing Enzymes for the Efficient Eradication of Oral Biofilms
Source: Chem Mater. 2024 Sep 30;36(19):9860–73. doi: 10.1021/acs.chemmater.4c02014 (PMC11468777; doi:10.1021/acs.chemmater.4c02014)
Supplement: Supplementary file 1 — cm4c02014_si_001.pdf [file cm4c02014_si_001.pdf]

Supporting Information

Lysozyme-responsive Hydrogels of Chitosan-streptomycin Conjugates for the On-demand Release of Biofilm-dispersing Enzymes for the Efficient Removal of Oral Biofilms

María Luisa del Pozo, Antonio Aguanell, Eduardo García-Junceda, and Julia Revuelta*.*

BioGlycoChem Group. Departamento de Química Bio-Orgánica. Instituto de Química Orgánica General, CSIC (IQOG-CSIC). Juan de la Cierva 3, 28006 Madrid, Spain.

1. Selection of starting chiosan.

Previous studies have shown that the rate of chitosan degradation with Lyz depends on the different structural variables that define chitosan, such as molecular weight, degree of acetylation or crystallinity.[1–3]

On this basis, we selected five chitosan polysaccharides with different structural properties **(Table S1)**. On the one hand, we analyzed three commercial chitosan samples with similar deacetylation degree but with significant differences in molecular weight (CS-1, CS-2 and CS-3). In addition, the influence of the degree of acetylation was analyzed by comparing CS-3 and its re-acetylated product CS-5.[4] Finally, to determine the possible influence of crystallinity, a chitosan sample obtained from squid pen chitin (CS-4) was also analyzed. In this context, it is important to note that chitosan has different polymorphic forms depending on its origin, which are related to its degradability mediated by Lyz.[5] In general, α-chitosan is obtained from chitin isolated from crustaceans, while β-chitosan is obtained from non-conventional sources such as squid pen.

Table S1. Structural properties of analyzed chitosan samples.

CHITOSAN		Crystallinity of starting chitin	Deacetylation degree (%)	Mw (kDa)[1]
Commercial chitosan samples	CS-1	α-chitin	5.00 ± 0.01	50 ± 2
	CS-2		23.00 ± 0.05	91.5 ± 2
	CS-3		15.00 ± 0.01	125 ± 5
Non-commercial chitosan sample	Squin-pen chitosan (CS-4)	β-chitin	5.93 ± 0.01	153 ± 3
Re-acetylated chitosan	CS-3-Ac (CS-5)	α-chitin	35.00 ± 0.05	127 ± 3

[1] Mw: weight average molar mass;

The analysis of reducing sugars by Park-Johnson assay has been used as an indirect method to determine the rate of chitosan degradation, since these reducing sugars are formed by the

enzymatic cleavage of the glycosidic bond between two glucosamine-chitosan units.[6] The reductive ends of chitosan were measured in the first three hours after addition of Lyz and after 24 hours **(Figure S1)**. At this point, new Lyz was added to the reaction mixtures, after which the reaction progress was monitored for three additional hours.

In all cases, the rate of degradation mediated by Lyz was low, which is sufficient for the required long-term availability of the hydrogel. However, differences in the mode of degradation were observed. To obtain a continuous release profile, Lyz-mediated degradation of chitosan must be gradual to allow controlled release of chitosan fragments over time. In this sense, the best results were obtained with chitosan CS-1, which degrades gradually even after the second addition of Lyz, which is why it was chosen as the starting material for hydrogel development.

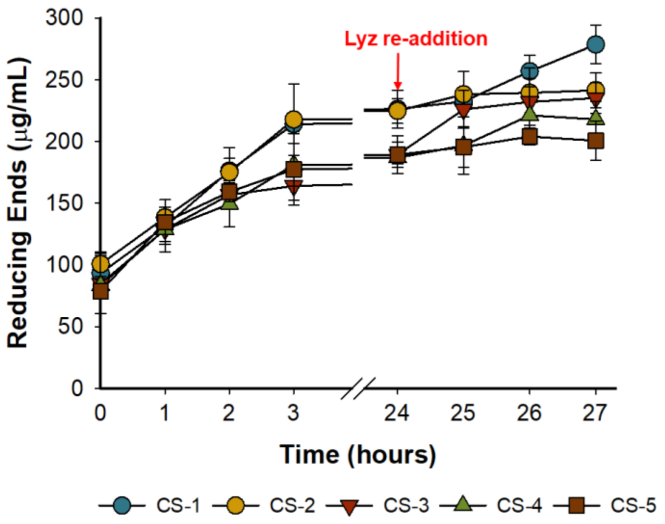

Figure S1. *In vitro* analysis of the rate of degradation of chitosan samples with Lyz.

2. Experimental protocols

2.2.1. Determination of chitosan acetylation degree.

The degree of acetylation (DA) was calculated from 1H NMR according to the method described by Jiang et al. (2017)[7], using eq S1:

$$DA(\%) = \frac{3xA_2}{6xA_1} \times 100 \qquad \text{(S1)}$$

where A1 are the protons integral values of positions C2–C6 on the sugar ring and A2 are the protons integral values of the *N*-acetyl protons of the *N*-acetyl-D-glucosamine units.

***2.2.2. Measurement of lysozyme activity by determination of reducing sugars using the Park Johnson assay*[8].** 4 mg of each chitosan (CS-1, CS-2, CS-3 or CS-4) was dissolved in 1 mL of 50mM acetate/acetic acid buffer (pH=4) and 250 µL of this solution was taken and diluted in 650 µL of the same buffer to which 100 µL of a solution of lysozyme at a concentration of 6 mg/mL was added. Incubation was carried out at 40 ºC with gentle shaking, and 200 µL aliquots were withdrawn after 60, 120 and 180 minutes and after 24 hours. The reaction was stopped by heating at 95 ºC for 5 minutes and centrifuging at 12,000 g for 10 minutes.

The reducing ends of the chitosan, which remain free after digestion with lysozyme, were measured in the supernatant, as these aldehyde groups are susceptible to oxidation. Therefore, we use a modification of the Park-Johnson method for their determination, which is based on a redox reaction between the free aldehyde groups and the potassium ferrocyanide of the reagent. For this purpose, 222 µl of alkali solution (1.5 mM potassium ferrocyanide, 50 mM sodium carbonate and 10 mM potassium cyanide) was added to 111 µl of the supernatant and the reaction was started by heating the mixture to 95 ºC for 15 minutes. After cooling to room temperature, 555 µL of an iron(III) ammonium sulfate solution (1.36 mM iron(III) ammonium sulfate dissolved in 25 mM sulfuric acid) was added and heated again to 50 ºC for 15 minutes, forming a blue complex called Prussian blue. Finally, after cooling to room temperature, 111 µl of a 125 mM oxalic acid solution was added, which reduced the excess iron left released in the previous step without reaction, and after stirring, the absorbance was measured at 690 nm. The concentration of reducing-ends was calculated using a *D*-glucosamine standard curve. The increase of reducing-ends was calculated according using **eq S2**:

$$\Delta\ [reducing - ends]\ (\%) = \frac{Cf - Ci}{Ci} \times 100 \qquad \text{(S2)}$$

where *Ci* and *Cf* indicate the reducing end concentration at the beginning and at the end of the experiment.

3. Supplementary figures

Figure S2. ^{1}H NMR of Str (top) and CS-Str (DS= 16.8%) conjugate. Signals that disappear in the spectrum of the streptomycin conjugate are indicated by a red box.

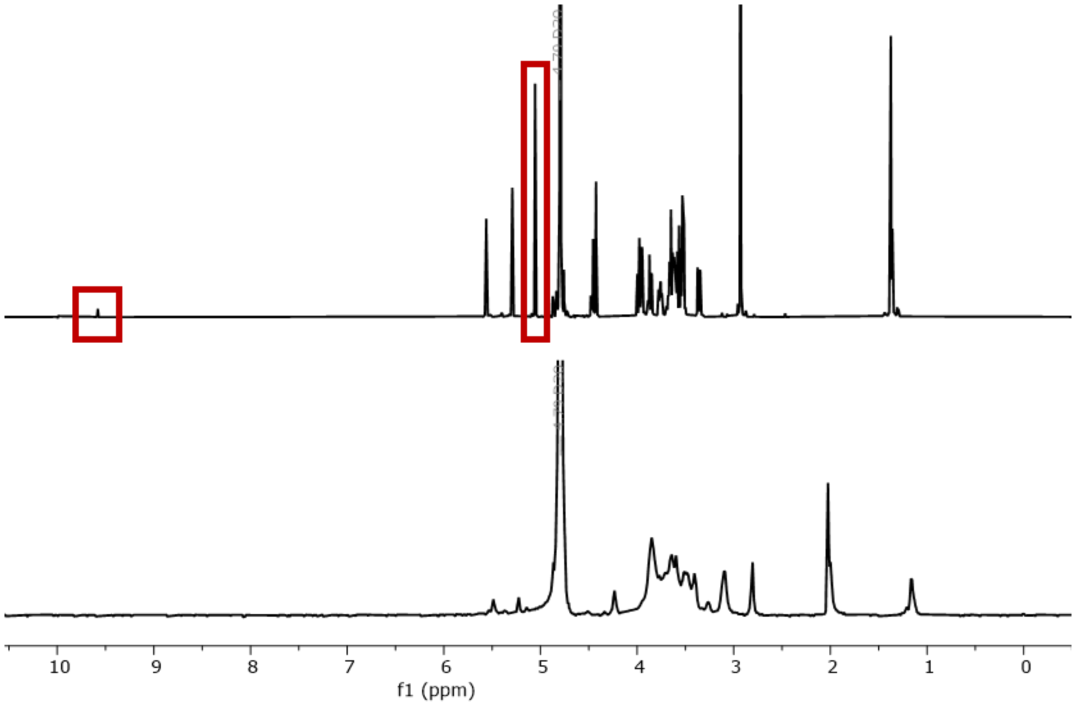

Figure S3. ^{1}H NMR CS-Str. The overall degree of streptomycin incorporation (DSI) was determined by comparing the ^{1}H-NMR integrals of the protons of the methyl group of the streptomycin molecule (1.22 ppm) with respect to the protons of the acetyl group of the chitosan molecule (2.01 ppm)

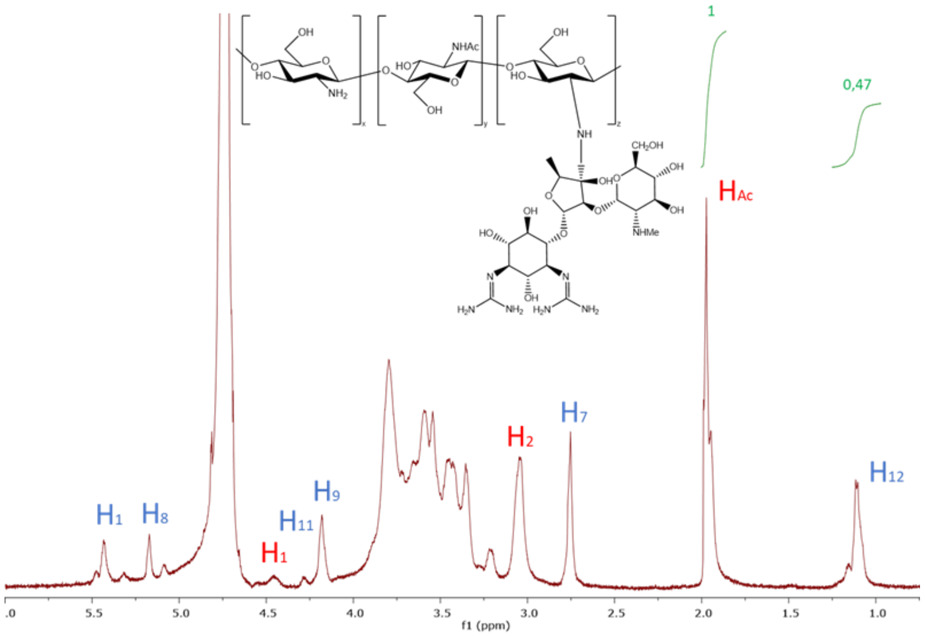

Figure S4. Representative photographs of plate bacterial count of hydrogels and biofilm for *P. aeruginosa* (NCIMB 8295) (A) and *S. aureus* (NCTC 8532) (B).

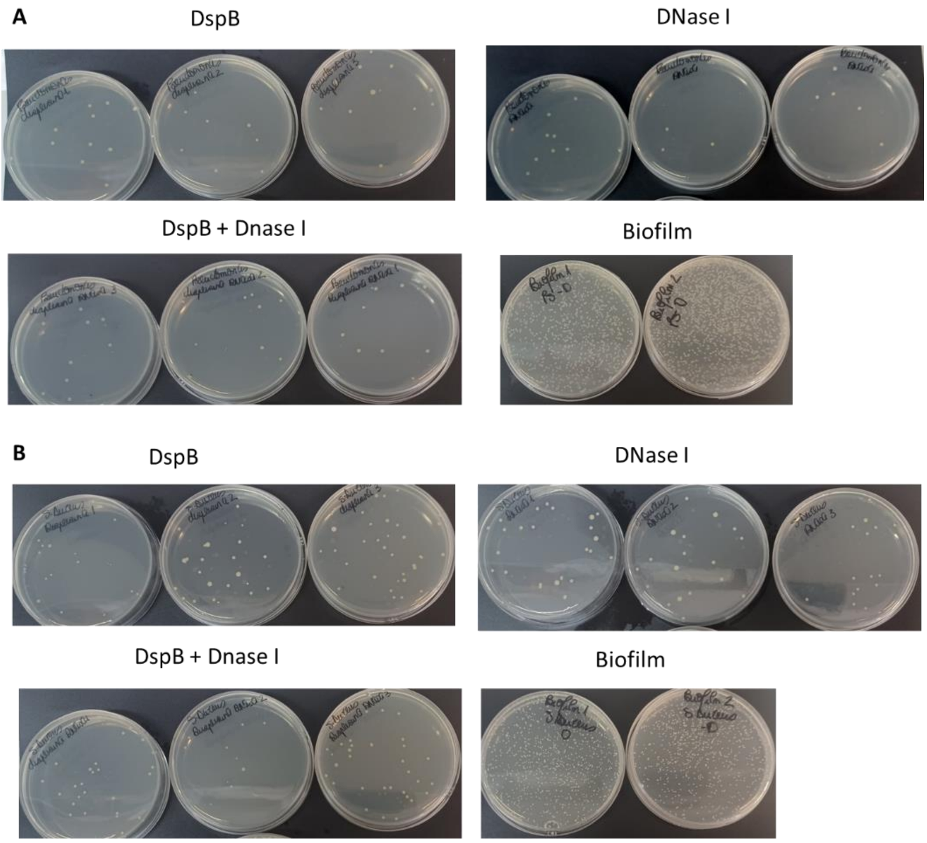

References

(1) Kean, T.; Thanou, M. Biodegradation, Biodistribution and Toxicity of Chitosan. *Adv. Drug Deliv. Rev.* **2010**, *62* (1), 3–11. https://doi.org/https://doi.org/10.1016/j.addr.2009.09.004.

(2) Lončarević, A.; Ivanković, M.; Rogina, A. Lysozyme-Induced Degradation of Chitosan: The Characterisation of Degraded Chitosan Scaffolds. *J. Tissue Repair Regen.* **2017**, *1* (1), 12–22. https://doi.org/10.14302/issn.2640-6403.jtrr-17-1840.

(3) Vårum, K. M.; Myhr, M. M.; Hjerde, R. J. N.; Smidsrød, O. In Vitro Degradation Rates of Partially N-Acetylated Chitosans in Human Serum. *Carbohydr. Res.* **1997**, *299* (1), 99–101. https://doi.org/https://doi.org/10.1016/S0008-6215(96)00332-1.

(4) Revuelta, J.; Aranaz, I.; Acosta, N.; Civera, C.; Bastida, A.; Peña, N.; Monterrey, D. T.; Doncel-Pérez, E.; Garrido, L.; Heras, Á.; García-Junceda, E.; Fernández-Mayoralas, A. Unraveling the Structural Landscape of Chitosan-Based Heparan Sulfate Mimics Binding to Growth Factors: Deciphering Structural Determinants for Optimal Activity. *ACS Appl. Mater. Interfaces* **2020**, *12* (23), 25534–25545. https://doi.org/10.1021/acsami.0c03074.

(5) Bagheri-Khoulenjani, S.; Taghizadeh, S. M.; Mirzadeh, H. An Investigation on the Short-Term Biodegradability of Chitosan with Various Molecular Weights and Degrees of Deacetylation. *Carbohydr. Polym.* **2009**, *78* (4), 773–778. https://doi.org/https://doi.org/10.1016/j.carbpol.2009.06.020.

(6) Thompson, J. S.; Shockman, G. D. A Modification of the Park and Johnson Reducing Sugar Determination Suitable for the Assay of Insoluble Materials: Its Application to Bacterial Cell Walls. *Anal. Biochem.* **1968**, *22* (2), 260–268. https://doi.org/https://doi.org/10.1016/0003-2697(68)90315-1.

(7) Jiang, Y.; Fu, C.; Wu, S.; Liu, G.; Guo, J.; Su, Z. Determination of the Deacetylation Degree of Chitooligosaccharides. *Mar. Drugs* **2017**, *15* (11), 332. https://doi.org/10.3390/md15110332.

(8) Park, J. T.; Johnson, M. J. A Submicrodetemination of Glucose. *J. Biol. Chem.* **1949**, *181* (1), 149–151.
